# Distinct Longitudinal Changes in EEG Measures Reflecting Functional Network Disruption in ALS Cognitive Phenotypes

**DOI:** 10.1007/s10548-024-01078-8

**Published:** 2024-10-04

**Authors:** Marjorie Metzger, Stefan Dukic, Roisin McMackin, Eileen Giglia, Matthew Mitchell, Saroj Bista, Emmet Costello, Colm Peelo, Yasmine Tadjine, Vladyslav Sirenko, Lara McManus, Teresa Buxo, Antonio Fasano, Rangariroyashe Chipika, Marta Pinto-Grau, Christina Schuster, Mark Heverin, Amina Coffey, Michael Broderick, Parameswaran M. Iyer, Kieran Mohr, Brighid Gavin, Niall Pender, Peter Bede, Muthuraman Muthuraman, Orla Hardiman, Bahman Nasseroleslami

**Affiliations:** 1 Academic Unit of Neurology, School of Medicine, Trinity Biomedical Sciences Institute, Trinity College Dublin, University of Dublin, Room 5.43, 152–160 Pearse Street, Dublin 2, D02 R590 Ireland; 2 Department of Neurology, University Medical Centre Utrecht Brain Centre, Utrecht University, Utrecht, 3584 CG The Netherlands; 3 Discipline of Physiology, School of Medicine, Trinity Biomedical Sciences Institute, Trinity College Dublin, University of Dublin, Dublin 2, D02 R590 Ireland; 4 Computational Neuroimaging Group, Trinity Biomedical Sciences Institute, Trinity College Dublin, University of Dublin, D02 R590 Dublin 2, Dublin 2, Ireland; 5 Trinity Centre for Bioengineering, Trinity College Dublin, University of Dublin, D02 R590 Dublin 2, Dublin 2, Ireland; 6 Neural Engineering with Signal Analytics and Artificial Intelligence, Department of Neurology, University Hospital Würzburg, 97080 Würzburg, Germany; 7 Trinity College Institute of Neuroscience, Trinity College Dublin, University of Dublin, Dublin 2, D02 PN40 Ireland; 8 Beaumont Hospital, D09 V2N0 Dublin 9, Dublin, Ireland; 9 FutureNeuro - SFI Research Centre for Chronic and Rare Neurological Diseases, Royal College of Surgeons, D02 YN77 Dublin 2, Dublin 2, Ireland

**Keywords:** Neurodegeneration, Spectral resting-state EEG, Source localisation, Motor neuron disease, Cognitive-behavioural impairments, Functional connectivity

## Abstract

Amyotrophic lateral sclerosis (ALS) is characterised primarily by motor system degeneration, with clinical evidence of cognitive and behavioural change in up to 50% of cases. We have shown previously that resting-state EEG captures dysfunction in motor and cognitive networks in ALS. However, the longitudinal development of these dysfunctional patterns, especially in networks linked with cognitive-behavioural functions, remains unclear. Longitudinal studies on non-motor changes in ALS are essential to further develop our understanding of disease progression, improve care and enhance the evaluation of new treatments. To address this gap, we examined 124 ALS individuals with 128-channel resting-state EEG recordings, categorised by cognitive impairment (ALSci, *n* = 25), behavioural impairment (ALSbi, *n* = 58), or non-impaired (ALSncbi, *n* = 53), with 12 participants meeting the criteria for both ALSci and ALSbi. Using linear mixed-effects models, we characterised the general and phenotype-specific longitudinal changes in brain network, and their association with cognitive performance, behaviour changes, fine motor symptoms, and survival. Our findings revealed a significant decline in $\:\theta\:$-band spectral power over time in the temporal region along with increased $\:{\gamma\:}_{l}$-band power in the fronto-temporal region in the ALS group. ALSncbi participants showed widespread β-band synchrony decrease, while ALSci participants exhibited increased co-modulation correlated with verbal fluency decline. Longitudinal network-level changes were specific of ALS subgroups and correlated with motor, cognitive, and behavioural decline, as well as with survival. Spectral EEG measures can longitudinally track abnormal network patterns, serving as a candidate stratification tool for clinical trials and personalised treatments in ALS.

**Supplementary Information:**

The online version contains supplementary material available at 10.1007/s10548-024-01078-8.

## Introduction

Amyotrophic lateral sclerosis (ALS) is a progressive, neurodegenerative disease, which affects upper and lower motor neurons. In addition to changes in the motor pathways, ALS is associated with widespread alterations in extra-motor cortical regions and has links to frontotemporal dementia (FTD) (Swinnen and Robberecht 2014). Cognitive and/or behavioural abnormalities are present in up to 40% of people with ALS, while an additional 14% develop dementia (Pender et al. 2020; Phukan et al. 2012). The presence of such a range of clinical characteristics in the disease leads to uncertainties when diagnosing ALS and predicting its course. There is therefore an urgent need for robust and validated phenotypic biomarkers to better predict disease progression and responses to potential therapies (Bede and Hardiman 2018; Nasseroleslami 2018; Pender et al. 2020; Taga and Maragakis 2018).

Neuroimaging methods such as electrophysiology, functional and structural magnetic resonance imaging (fMRI-sMRI) have played a crucial role in identifying brain alterations in ALS. These modalities have shown variations in grey matter and functional connectivity within distinct ALS cognitive-behavioural profiles and in behavioural (bv-)FTD, both cross-sectionally (Govaarts et al. 2022; Temp et al. 2021) and longitudinally (Burgh et al., 2020; Shen et al. 2018). Recent studies using EEG to directly measure and quantify the function of brain networks, including those underlying cognition and behaviour, have shown that such neuroimaging methods have great promise in the search for ALS biomarkers (McMackin et al. 2019, 2020).

Resting-state electroencephalography (EEG) emerges as a particularly accessible and participant-friendly method compared to tasks that require specific actions or responses, mitigating potential biases associated with speech or motor disabilities (Maruyama et al. 2021; Secco et al. 2020). Notably, longitudinal studies have identified persistent differences between participants with ALS and HC in resting-state EEG measures of neural activity ($\:\theta\:$-band spectral power) and functional connectivity ($\:\theta\:$, $\:{\gamma\:}_{h}$-band coherence) (Nasseroleslami et al. 2019). Source localisation of EEG signals has further elucidated disease-specific patterns in brain activity by determining the specific brain regions that generated the electrical activities recorded on the scalp (Michel and Brunet 2019).

Specifically, decreased neural activity across different frequency bands has been observed in motor and non-motor networks in ALS. Decreased synchrony between signals has also been noted in the frontotemporal and sensorimotor regions, while increased co-modulation (amplitude envelope correlation) of signals has been observed in central, posterior, and frontal areas (Dukic et al. 2019). Increased functional connectivity has been similarly observed in posterior regions using RS magnetoencephalography (MEG) (Proudfoot et al. 2018). The EEG functional connectivity changes correlated with MRI structural atrophy observed in cognitive networks, specifically within the frontal region. Additionally, these changes aligned with clinical assessments of cognitive function (Dukic et al. 2019). This suggests a cohesive relationship between EEG patterns, structural changes in the brain, and the clinical manifestation of cognitive abilities.

Building on our prior research, which identified four distinct patterns of network dysfunction using resting-state EEG (Dukic et al. 2022), this longitudinal study aims to validate the reliability and effectiveness of EEG measures in quantifying network-level impairment in ALS. Our primary objective is to identify and quantify changes in functional brain networks as ALS progresses, revealing how distinct cognitive phenotypes of ALS manifest unique patterns of longitudinal network impairment. Our secondary objective is to explore how longitudinal network changes correlate with clinical progression and survival in ALS. We assessed whether these changes reflect the progression in symptomatic impairments by examining their association with survival duration and disease progression, through functional – ALS Functional Rating Scale-Revised, ALSFRS-R (Cedarbaum et al. 1999) –, cognitive – Edinburgh Cognitive and Behavioral ALS Screen, ECAS (Abrahams et al. 2014) – and behavioural scores – Beaumont Behavioral Inventory, BBI (Elamin et al. 2017).

## Materials and Methods

### Ethical Approval

The study was approved by the Tallaght University Hospital / St. James’s Hospital Joint Research Ethics Committee - Dublin [references: 2014 Chairman’s Action 7; 2019-05 List 17 (01)] and performed in accordance with the Declaration of Helsinki. All participants provided informed written consent to the procedures before undergoing assessment.

### Participants

Recruitment of individuals with ALS occurred in the Irish National ALS Clinic in Beaumont Hospital, Dublin, Ireland. Individuals with Suspected to Definite ALS (El Escorial criteria; Suspected: *n* = 1, Possible: *n* = 26, Probable: *n* = 32, Lab supported probable: *n* = 8, Definite: *n* = 43) and with ALS-frontotemporal dementia (ALS-FTD: *n* = 5) diagnoses were included. All individuals with ALS-FTD included in this study were diagnosed with the behavioural variant.

Individuals without impairment of both upper and lower motor neurons were excluded (diagnosed as primary lateral sclerosis (PLS), progressive muscular atrophy (PMA), or flail arm/leg syndromes), as well as individuals with other medical morbidities or neurological abnormalities.

### Experiment

#### Experimental Design

The recruited participants attended EEG recording sessions in the Clinical Research Facility of Saint James Hospital (n.d.), Dublin. No blinding was performed, as the participants, experimenters or data analysts could not access the final EEG measures during experiments. EEG data from 124 individuals with ALS (male: 69.3%; age [mean ± standard deviation]: 63.13 ± 15) were recorded. The study included up to four follow-up recording sessions, with approximately 5.4 ± 2.1 months between sessions. The total number of EEG recordings was 249, of which 116 were baseline and 60, 44, 22 and 7 were follow-up 1–4, respectively. On average, the participants attended a total of 2 ± 1.2 recording sessions. Table 1 details the demographic profile for each follow-up.

**Table 1:** Demographic profiles of the individuals with ALS

Group	*N*	M (%)	Age (years)	Disease duration [increment since T1] (months)	Follow-up interval (months)	ALSFRS-*R* score (at Tx)	Site of onset (*N*)			ALS-FTD diagnosis (*N*)
							Bulbar	Spinal	Thoracic	
T1	116	74	62 ± 11	25 ± 18	/	36 ± 7	22 (19%)	86 (74%)	5 (4%)	5
T2	60	77	60 ± 11	32 ± 19 [+ 7.3]	4.9 ± 1.2	35 ± 8	14 (23%)	43 (72%)	2 (3%)	2
T3	44	80	60 ± 12	37 ± 19 [+ 12]	4.9 ± 1.3	33 ± 9	8 (18%)	34 (77%)	1 (2%)	2
T4	22	86	61 ± 11	42 ± 24 [+ 17]	4.9 ± 1.1	33 ± 7	2 (9%)	19 (86%)	1 (1%)	1
T5	7	57	57 ± 13	52 ± 31 [+ 27]	6.5 ± 2.4	33 ± 6	0	6 (86%)	1 (14%)	0

Up to five recording sessions were scheduled, with in-between time delays representing delays between each session. The table details the gender proportions (percentage of males), the average ages at recording and, when applicable, disease durations, delays between sessions, site of onset and the number of patients with FTD comorbidity for each recording timepoint (T1-T5). Numbers show mean and standard deviation

#### EEG Acquisition

The experiments were conducted at the Clinical Research Facility (CRF) of St James’s Hospital, Dublin using a 128-channel Biosemi ActiveTwo system (Honsbeek et al. 1998). The data were recorded while participants were at rest with eyes open, seated approximately 1 m from a letter X (6x8cm^2^), used as a target to focus their gaze on. The EEG signals were recorded in three blocks of 2 min, at 512 Hz with a low-pass anti-aliasing filter (cut-off at 104 Hz).

#### Disease Severity and Neuropsychology Assessment

Disease severity was assessed using ALSFRS-R scores (Cedarbaum et al. 1999) and King’s staging system (Balendra et al. 2014), collected from the Irish Motor Neuron Disease Registry. The participants’ ALSFRS-R scores were recorded on average 7.4 ± 5.1 times, between 3.7 and 145 months after symptom onset. The symptoms were evaluated at intervals of approximately 3.3 ± 3.7 months. Except for 9 participants from the EEG database, all participants had their ALSFRS-R scores registered. The scale consists of 12 scores of common task performance, rated between 0 and 4 (normal functioning). The scores are summed to produce an overall score between 0 and 48. To be anatomically relevant, subgroups of the total ALSFRS-R score were extracted and defined as ‘bulbar’ (scores 1–3), ‘upper limbs’ (scores 4–6), ‘lower limbs’ (scores 7–9) and ‘respiratory’ (scores 10–12), respectively for tasks falling within the bulbar region, the upper/lower limbs or the respiratory system. Survival times, calculated from symptom onset, were also collected from the Irish Motor Neuron Disease Registry.

To provide wider clinical profiling, the Edinburgh Cognitive and Behavioral ALS Screen (ECAS) (Abrahams et al. 2014) and Beaumont Behavioural Inventory (BBI) (Elamin et al. 2017), which were developed to compensate for the impact of motor impairment in ALS, were also obtained from parallel ongoing research projects in the Academic Unit of Neurology (Costello et al. 2020, 2021). The ECAS cognitive subscores (language, fluency, executive, memory and visuospatial) were obtained up to three times, with a total ECAS score range of 46 to 135. The abnormality cut-off was adapted for age and level of education. Three versions of the ECAS (A, B and C) were used to reduce practice effects (Costello et al. 2020; Crockford et al. 2018). Behaviour was assessed using the Beaumont behavioural inventory. BBI assessments were conducted up to three times for each participant, between 2.8 and 100 months after onset, yielding scores ranging from 0 to 73 (above 6 representing mild impairment and above 22 representing severe impairment).

### Subgrouping of Participants According to Their Cognitive-Behavioural Profiles

To analyse the effect of the cognitive and behavioural impairment on spectral EEG measures, the longitudinal trajectories were modelled separately for subgroups of individuals with ALS: cognitively impaired (ci), behaviourally impaired (bi) and non-impaired (ncbi). This discrimination by neuropsychological profiles was included with the expectation that participants with cognitive or behavioural impairment experience different progressions of neurodegeneration compared to participants with normal cognition and behaviour. The ALS group comprised a total of 124 participants, with 25 participants exhibiting cognitive impairment (ALSci), 58 participants demonstrating behavioural impairment (ALSbi), and 53 participants showing no cognitive or behavioural impairment (ALSncbi). 12 participants were part of both the ALSci and ALSbi groups due to displaying both cognitive and behavioural impairments. Participants were considered to have abnormal cognition if their ECAS scores exceeded the abnormality cut-off scores based on age and education for the Irish population (Pinto-Grau et al. 2017; Crockford et al. 2018; Costello et al. 2020). The assessment of abnormal behavior was conducted using the BBI, with behavioural impairment defined as scoring ≥ 6 points on the BBI scale.

### Data Analysis

#### Pre-Processing

The EEG signals were pre-processed using MATLAB (version R2019b) (The MathWorks 2019), the EyeBallGUI toolbox (Mohr et al. 2017) and the Fieldtrip v20190905 toolbox (Oostenveld et al. 2011). An automatic artefact rejection method was used to reject bad epochs in the EEG signals (Dukic et al. 2017). For this purpose, the amplitude, the mean shift, the variance and the band-variance of spectral power were checked against a 3.5 Z-score threshold. The EEG signal was resampled at 256 Hz, band-pass (one-pass zero-phase FIR: 1–97 Hz) and notch filtered (third-order Butterworth: 50 Hz). An automatic algorithm, which evaluates the correlation between EEG channels, high signal standard deviations and the ratio of high to low frequencies, was then used to detect noisy channels (Bigdely-Shamlo et al. 2015; Kohe 2010). The average number of removed channels was 3.9 ± 8.6. If more than 11 channels were marked as noisy, the recording was excluded from the study. Channels that were marked for removal were interpolated from the remaining electrodes using spline interpolation (Oostenveld et al. 2011). Finally, the channels were referenced to the common average.

#### Processing

The EEG data were processed as described by Dukic et al. (Dukic et al. 2019). Namely, source localisation was performed using the Linearly Constrained Minimum Variance (LCMV) beamformer (Oostenveld et al. 2011) and a head model based on the ICBM152 MRI template (Fonov et al. 2009). The source-space signals were estimated in 90 brain regions from the automated anatomical labelling (AAL) atlas (Tzourio-Mazoyer et al. 2002). Using these source-localised signals, spectral measures were computed in six frequency bands usually considered in resting-state EEG studies (Dukic et al. 2017; Iyer et al. 2015), i.e. $\:\delta\:$ (2–4 Hz), $\:\theta\:$ (4–7 Hz), $\:\alpha\:$ (7–13 Hz), $\:\beta\:$ (13–30 Hz), $\:{\gamma\:}_{l}$ (30–47 Hz) and $\:{\gamma\:}_{h}$ (53–97 Hz). For each brain region, normalised spectral power was estimated using a Fast Fourier analysis applied on 2 s epochs. For all pairs of brain regions, functional connectivity was estimated using amplitude envelope correlation ($\:AEC$) corrected for spatial leakage (Brookes et al. 2012), which measures the co-modulation, and using imaginary coherence (iCoh), which measures the synchrony between two regions of interest (ROIs).

#### Statistical Analysis

Linear mixed-effects (LME) models, which can account for heterogeneous progressions of disease, were used to track the changes in the brain network patterns of participants over time. This method has been shown to be robust for sparse and unbalanced data sets and useful for longitudinal studies with missing data (West et al. 2007). LME Models were built to estimate the progressions of the EEG measures (normalised spectral power and connectivity) and of the clinical scores (ALSFRS-R, ECAS and BBI) over time.

##### Electroencephalography

Spectral power, co-modulation and synchrony were analysed separately for each frequency band ($\:\delta\:,\:\theta\:,\:\alpha\:,\beta\:,\:{\gamma\:}_{l},\:{\gamma\:}_{h}$). All LME models were built in MATLAB using the *fitlme* function with the Quasi-Newton method as the iterative algorithm for data fitting and optimising the likelihood function and Restricted Maximum Likelihood to avoid bias in the estimated covariance parameters. F-statistics were conducted to test for significant fixed-effects. The purpose of our LME models was to check whether there is an overall main effect of time (in the entire brain) but also to estimate the rate of progression in each brain region (modelled as random-effects and used subsequently for further analysis).

To reduce the dimensionality and avoid over-parameterisation (when not all the linear combinations of parameters are estimable), we regrouped and contracted the measures for broader brain areas: frontal, temporal, motor, parietal, occipital and subcortical areas (see Supplementary note S1: brain networks). The subgrouping was initially based on the five anatomical lobes: frontal, temporal, centro-parietal, occipital and subcortical. Since the motor cortex is a key area of atrophy in ALS, the centro-parietal lobe was subsequently separated into the parietal lobe and the motor network.

To assess where significant longitudinal changes occurred, a bootstrapping method was performed on ROI-specific LME models (1 model per ROI): Measure ~ Time + (Time|Participant). To evaluate the null-hypothesis of no time effect on the EEG measures, the (two to five) timepoints were randomly resampled in each participant before computing a new LME model (1000 repetitions). The results of this statistical method were then corrected to account for the number of ROIs using a 10% adaptive false discovery rate (FDR)(Benjamini et al. 2006; Nasseroleslami 2018) and applied as a mask to visualise estimated sources of neural activity changes over time.

Longitudinal trajectories of the EEG spectral power were estimated using the following model described in Wilkinson-Rogers notation:

Power ~ Time + (Time|Participant) + (Time|ROI).

The fixed-effects coefficient corresponds to the time since the onset of the disease (‘Time’). The random-effects include a participant-specific factor and an ROI-specific factor. Age and gender were considered for inclusion as random-effects but did not significantly improve the models (based on a likelihood ratio test) and hence were not included. The random-effect coefficients and the residuals associated with the EEG score of a participant, within a brain region, were checked to confirm they followed normal distributions, were independent, and had constant variance (using the Kolmogorov–Smirnov test (q < 0.05); Ljung-Box Q-test (q < 0.05); Engle’s ARCH test (q < 0.05) or diagnostic plots).

To estimate the connectivity progressions, we used a similar LME model with the same fixed-effects. It was then possible to add an interaction term to investigate participant-specific effects on the functional connectivity within or between brain regions of interest. In Wilkinson-Rogers notation, the model is described as:

Connectivity ~ Time + (Time|Participant) + (Time|ROI) + (-1 + Time|ROI: Participant).

All connectivity values were transformed using a rank-based inverse normal transformation (Beasley et al. 2009) to reduce the deviation of residuals from normality. Age and gender were again considered for inclusion as random-effects but did not significantly improve the models and therefore were not included.

##### Clinical Scores

After evaluating the progression of EEG measures, we analysed the evolution of functional, cognitive and behavioural clinical scores. We considered ALSFRS subscores as a measure of respiratory, bulbar and motor function; total ECAS scores and ECAS fluency subscores as a measure of cognitive impairment and BBI scores as a measure of behavioural change. The fluency subscore was specifically chosen because it has been reported as the cognitive subdomain that displays the most prominent and consistent impairment in ALS (Abrahams et al. 2000, 2014). A linear function was defined to represent each clinical score (ALSFRS-R subscores, total ECAS, ECAS fluency and BBI scores) progression over time. Each score was modelled as Score ~ Time + (Time|Participant). The fixed-effect coefficients can be described as the mean intercept and slope for all participants. Similarly, the random-effect coefficients described the participant-specific deviation from intercept and slope. Age, gender and education were considered for inclusion, but only education was deemed relevant for the ECAS models (based on a likelihood ratio test). For the cognitive progression models, an additional term was added to account for the versions of the ECAS questionnaire (A, B and C). This term was added despite the use of three alternate versions to reduce practice effects because some participants performed a sequence A-A-A, while others undertook sequence A-B-C, which needed to be addressed. The ECAS model can be described as: ECAS ~ Time + Version + (Time|Subj: Study) + (Time|Education). The assumptions of normality, independence, and constant variance of the residuals were checked (using the Kolmogorov–Smirnov test (q < 0.05); Ljung-Box Q-test (q < 0.05); Engle’s ARCH test (q < 0.05) or diagnostic plots).

##### Correlations between EEG and Clinical Measures

Following the estimation of both the linear mixed regressions of EEG measures and clinical scores (ALSFRS-R, ECAS, BBI) over time, the rank correlations between EEG measures and clinical score progressions were calculated. For each participant, the EEG longitudinal spectral measure changes at frequency f ($\:\delta\:$ to $\:{\gamma\:}_{h}$-bands), were estimated by the time-related participant-specific random-effect of the linear mixed model, which represents the slope or rate of change per month. Similarly, participant/ROI interactions showed participant-specific variations in specific brain regions. All random-effects slope signs were adjusted according to the fixed-effect slope (or according to the sum of the fixed-effect slope and ROI-specific slope in the case of a participant/ROI interactions) to represent faster or slower changes in regard to the average changes across participants. For each participant, the clinical changes ( ALSFRS-R bulbar, upper / lower limbs, respiratory, total ECAS, ECAS fluency or BBI) over time were also estimated by the time-related slope of the LME model.

Rank correlations were additionally calculated between EEG measures and survival.

Correlations between EEG and clinical measures were computed using the Spearman correlation coefficient. The statistical power of each correlation was estimated by bootstrapping (*N* = 2000) using the EBI toolbox (Nasseroleslami 2018). A 5% FDR correction (threshold chosen following the inspection of the Type-I -Type II relationship) was implemented for the multiple comparisons of the participant/ROI interactions correlating with clinical scores, separately for each frequency and each score of motor or cognitive decline. Correlations between EEG values and survival were corrected similarly with a 1% FDR to facilitate visualisation.

## Results

### Changes in Neural Activity: Decrease in slow Oscillations, Increase in Faster Oscillations

We observed distinct patterns of longitudinal changes in neurophysiological measures within the entire ALS group. As indicated above, the neurophysiological measures investigated were spectral power (intensity of oscillations in neuroelectric activity), AEC (co-modulation of signals, an amplitude-based measure of functional connectivity) and iCoh (synchrony between signals, a phase-based measure of functional connectivity between brain regions).

**Fig. 1:**
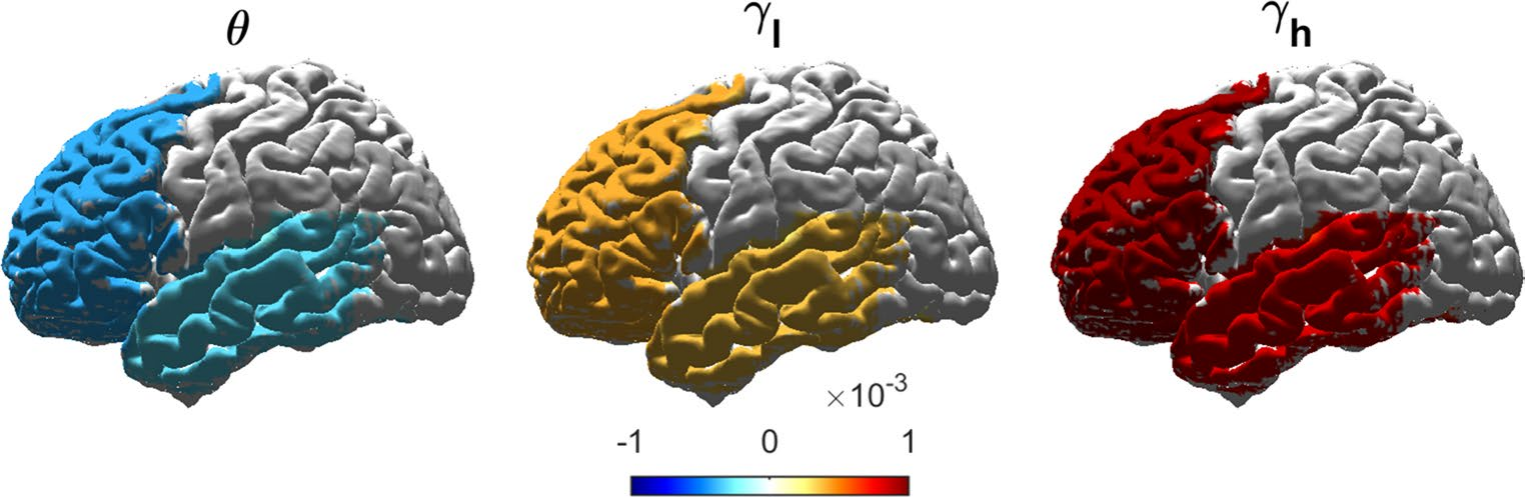
Changes in neural activity include a decrease in slow oscillations and increase in faster oscillations. Longitudinal changes of EEG spectral power in ALS were measured in term of significant longitudinal spectral power variations, based on the time fixed-effect and the time ROI-specific random-effects (Bootstrapping, *q* < 0.1). Longitudinal changes were mapped to get a spatial visualisation. The neural activity showed a significant decrease in $\:\theta\:$-band and an increase in $\:\gamma\:$-band

Across all participants, spectral power significantly increases over time in the frontal and temporal lobes in $\:\gamma\:$-band and decreases in $\:\theta\:$-band (Fig. 1). A detailed description of the models examining these effects can be found in Supplementary Note S2. In the following sections, the longitudinal effects in the ALSci, ALSbi, and ALSncbi subgroups were investigated to assess whether the observed frontotemporal network changes could have been driven by ALSbi or ALSci participants. A detailed description of the longitudinal effects per subgroup can be found in the Supplementary Material, Note S6.

### Longitudinal Changes in Functional Clinical Measures

We observed significant changes in longitudinal functional clinical scores, including ALSFRS-R and neuropsychological scores, within the entire ALS group. These scores serve as essential tools for assessing both the physical and cognitive progression of the disease using established qualitative measures as a reference. The models identified significant declines in ALSFRS-R scores (*p* < .001) and a slight increase in ECAS total scores (*p* < .05). Although some participants may exhibit a decrease in ECAS scores, 80% of the individuals in our dataset did not show any cognitive impairment at any point in time. Furthermore, verbal fluency and BBI scores did not exhibit significant changes over time, at group level. For a more detailed examination of the longitudinal models, please refer to Supplementary materials, Note S3.

### Widespread Increased EEG co-modulation in Cognitively Impaired Participants

**Fig. 2:**
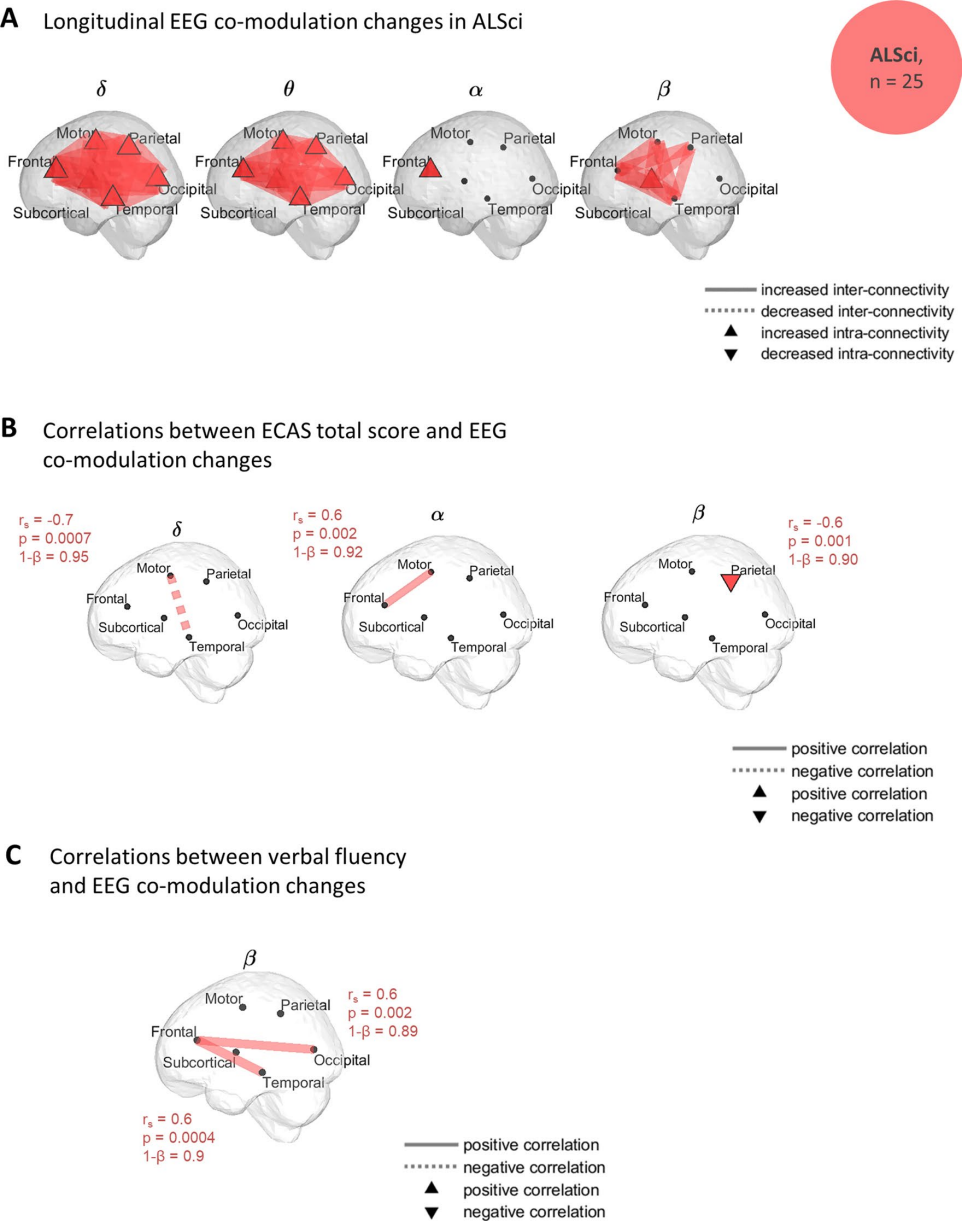
Widespread increased EEG co-modulation in ALSci, and associations with cognitive decline. (**A)** Regions of longitudinal changes of EEG co-modulation and synchrony in ALSci group. The significant longitudinal connectivity changes were mapped to get a spatial visualisation of their magnitudes. The longitudinal variations represent the combined estimated slope (significance by bootstrapping, *q* < 0.1). A widespread increase in $\:\delta\:$- and $\:\theta\:$-band co-modulation was observed in ALSci. The dashed lines represent a decrease while the solid lines represent an increase in connectivity. A filled node represents significant intra-region connectivity. (**B-C)** Regions with significant correlations between participant/ROI-specific co-modulation progressions and cognitive decline, in ALSci. For each significant correlation, the correlation coefficient, *r*_*s*_, the p-value, *p*, and the statistical power,1-*β* are given. An adaptive FDR was applied to Spearman’s correlation coefficients. **(B)** Correlations between EEG co-modulation and ECAS total score progressions. **(C)** Correlations between EEG co-modulation and ECAS verbal fluency changes. ALSci: individuals with ALS and impaired cognition; MM: intra-motor connectivity; MP: connectivity between motor and parietal regions; PP: intra-parietal connectivity; FO: connectivity between frontal and occipital regions; ST: connectivity between subcortical and temporal regions; SO: connectivity between subcortical and occipital regions

We observed widespread significant increased ($\:\delta\:$-, $\:\theta\:$- and $\:\beta\:$-band) co-modulation in ALSci (Fig. 2). The intra-frontal ($\:\alpha\:$-band) co-modulation also showed an increase for the ALSci subgroup. Significant functional connectivity changes observed between subsets of a specific region are referred as intra-regional connectivity (as opposed to inter-regional connectivity). A detailed description of the fixed-effects can be found in the Supplementary material, Note S2.

In ALSci, we observed that changes in participants’ region-specific connectivity were associated with corresponding neuropsychological changes, as assessed through three consecutive administrations of the ECAS (Fig. 2) (details in Supplementary material, Note S3). To elaborate further, higher rates of β-band co-modulation changes in connectivity between the frontal and occipital lobes, between the frontal and temporal lobes were found to be positively correlated with a more rapid decline in verbal fluency scores (correlation coefficients: *r*_*s*_ > 0.6, statistical powers: 1-$\:\beta\:$ > 0.89). Higher rates of changes in $\:\alpha\:$-band connectivity between the frontal and motor regions also positively correlated with a more rapid decline in fluency (correlation coefficients: *r*_*s*_ = 0.6, statistical powers: 1-$\:\beta\:$ = 0.92) By contrast, higher rates of changes either (i) within the parietal region (β-band) or (ii) between the motor and temporal lobes (δ-band) were associated with a decreased rate of change in cognition, affecting the ECAS total score (correlation coefficients: $\:\rho\:$ ≤ -0.6, statistical powers: 1-$\:\beta\:$ ≥ 0.90).

Significant associations were found between the co-modulation in ALSci and other clinical measures (bulbar and lower limbs ALSFRS-R subscores) and are detailed in Supplementary material, Note S8. No significant associations were found with BBI scores.

### Increased $\:{\gamma\:}_{l}$-band Spectral Power in Temporal lobe in Behaviourally Impaired Participants

**Fig. 3:**
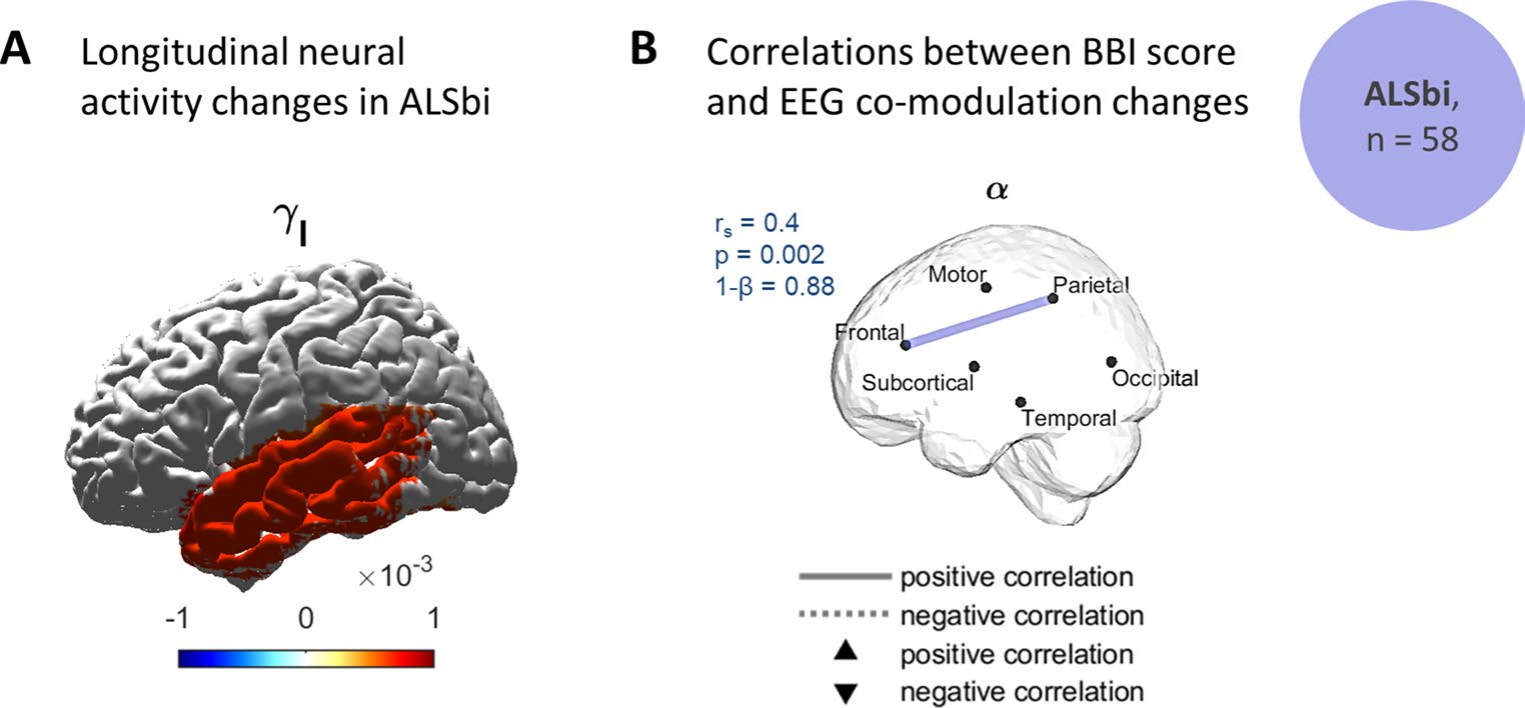
Increased EEG neural activity in the temporal lobe in ALSbi and associations with behavioural impairment. **(A)** Longitudinal changes of EEG spectral power in ALSbi. The significant temporal spectral power variations, in terms of the time fixed-effect and the time ROI-specific random-effects (Bootstrapping, *q* < 0.1), were mapped to get a spatial visualisation. An increase in $\:{\gamma\:}_{l}$-band co-modulation was observed in the temporal lobe for the ALSbi group. (**B)** Regions with significant correlations between participant/ROI-specific co-modulation progressions and cognitive decline, in the ALSbi group. A higher rate of change in co-modulation between the frontal and parietal lobes was correlated with an increased rate of change in BBI scores. For each significant correlation, the correlation coefficient, *r*_*s*_, the p-value, *p*, and the statistical power,1-*β*, are given. An adaptive FDR was applied to Spearman’s correlations. ALSbi: individuals with ALS and impaired behaviour

The spectral power significantly increased in the temporal lobe ($\:{\gamma\:}_{l}$-band) in ALSbi (Fig. 3). A detailed description of the fixed-effects can be found in the Supplementary material, Note S2.

Additionally, a higher rate of change in $\:\alpha\:$-band co-modulation between the frontal and parietal lobes was associated with an increased rate of changes in BBI scores (correlation coefficient: *r*_*s*_ =0.4, statistical powers: 1-$\:\beta\:$ =0.9). No significant associations were found between the co-modulation and other clinical measures (ALSFRS-R or ECAS scores) in ALSbi.

### Widespread Decreased β-band EEG Synchrony in Participants with Normal Cognition and Behaviour

**Fig. 4:**
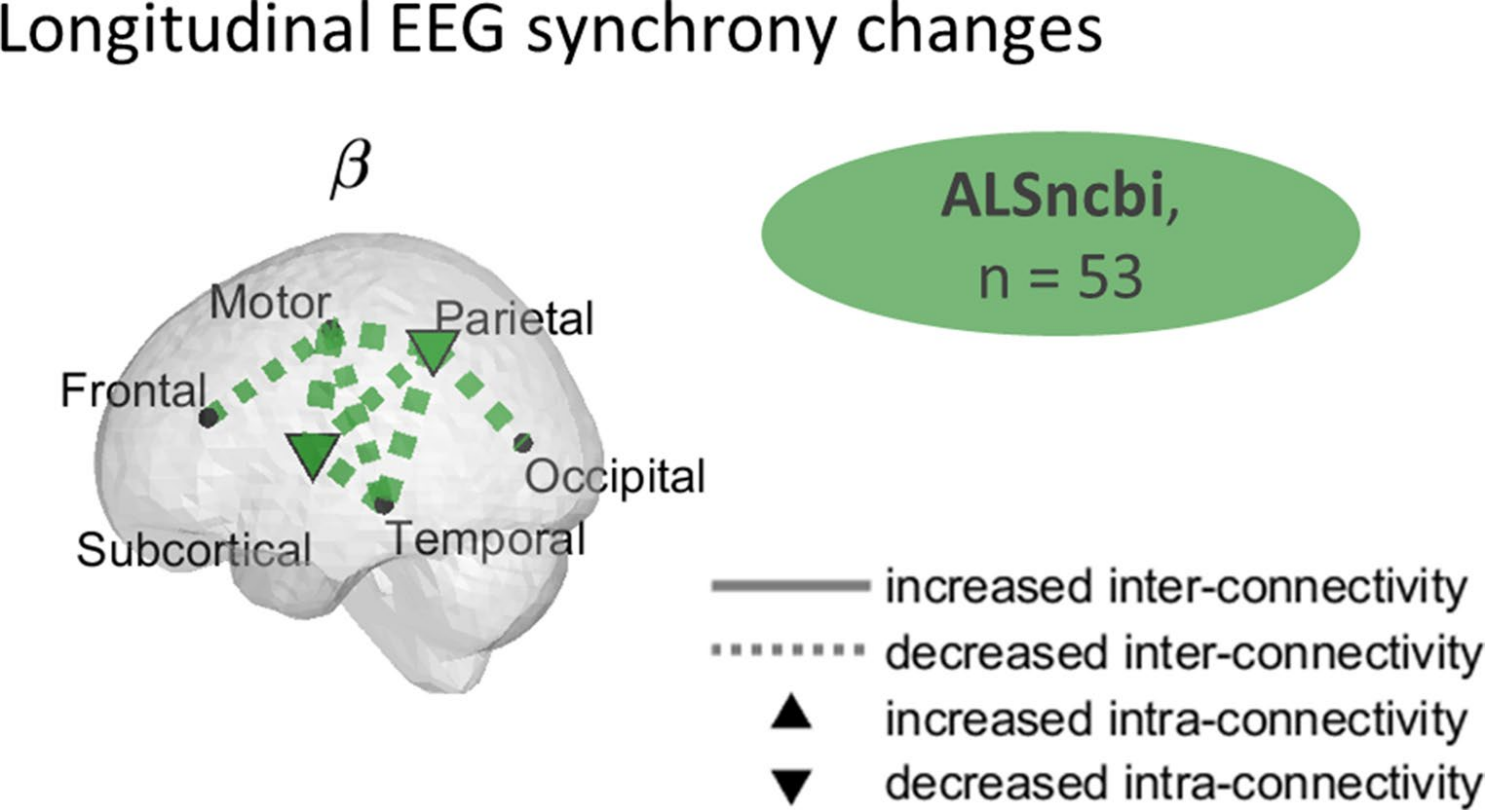
Widespread decreased $\:{\upbeta\:}$-band EEG synchrony in the ALSncbi group. Regions of longitudinal changes of EEG synchrony in the ALSncbi group. The significant longitudinal connectivity changes were mapped to get a spatial visualisation of their magnitudes. A widespread decrease in $\:{\upbeta\:}$-band synchrony was observed in the ALSncbi group. The longitudinal variations represent the combined estimated slope (significance by bootstrapping, *q* < 0.1). The dashed lines represent a decrease while the solid lines represent an increase in connectivity. A filled node represents significant intra-region connectivity. ALSncbi: individuals with ALS with normal cognition and behaviour

In ALSncbi participants, we observed widespread significant changes in synchrony, with an especially significant decrease in $\:\beta\:$-band (q < 0.1, Fig. 4). A detailed description of the fixed-effects, can be found in the appendix.

The slopes of the ALSFRS-R models (details in Supplementary material, Note S3) were used as an estimation of the speed of the disease progression per participant. We identified significant correlations of the changes in the clinical scores (progression rates) with the average brain-wide changes in neural activity (spectral power). In cognitively and behaviourally unaffected participants, the spectral power changes ($\:\theta\:$-, $\:{\gamma\:}_{l}$-, $\:{\gamma\:}_{h}$bands) negatively correlate (q < 0.05) with fine motor changes over time ($\:\theta\:$-band: $\:{r}_{s}\:$= -0.4, $\:p$ = 0.003, 1-β_0.05_ = 0.83; $\:{\gamma\:}_{l}$-band: $\:{r}_{s}\:$= -0.4, $\:p$ = 0.003, 1-β_0.05_ = 0.86; $\:{\gamma\:}_{h}$-band: $\:{r}_{s}\:$= -0.4, $\:p$ = 0.002, 1-β_0.05_ = 0.84). Higher rates of change in spectral power were associated with decreased rates of change in the fine motor score.

No significant associations were found between spectral power and the other clinical measures (other ALSFRS-R subscores or neuropsychological scores) in the ALSncbi group.

### Correlations between Survival and Changes in the EEG Measures

**Fig. 5:**
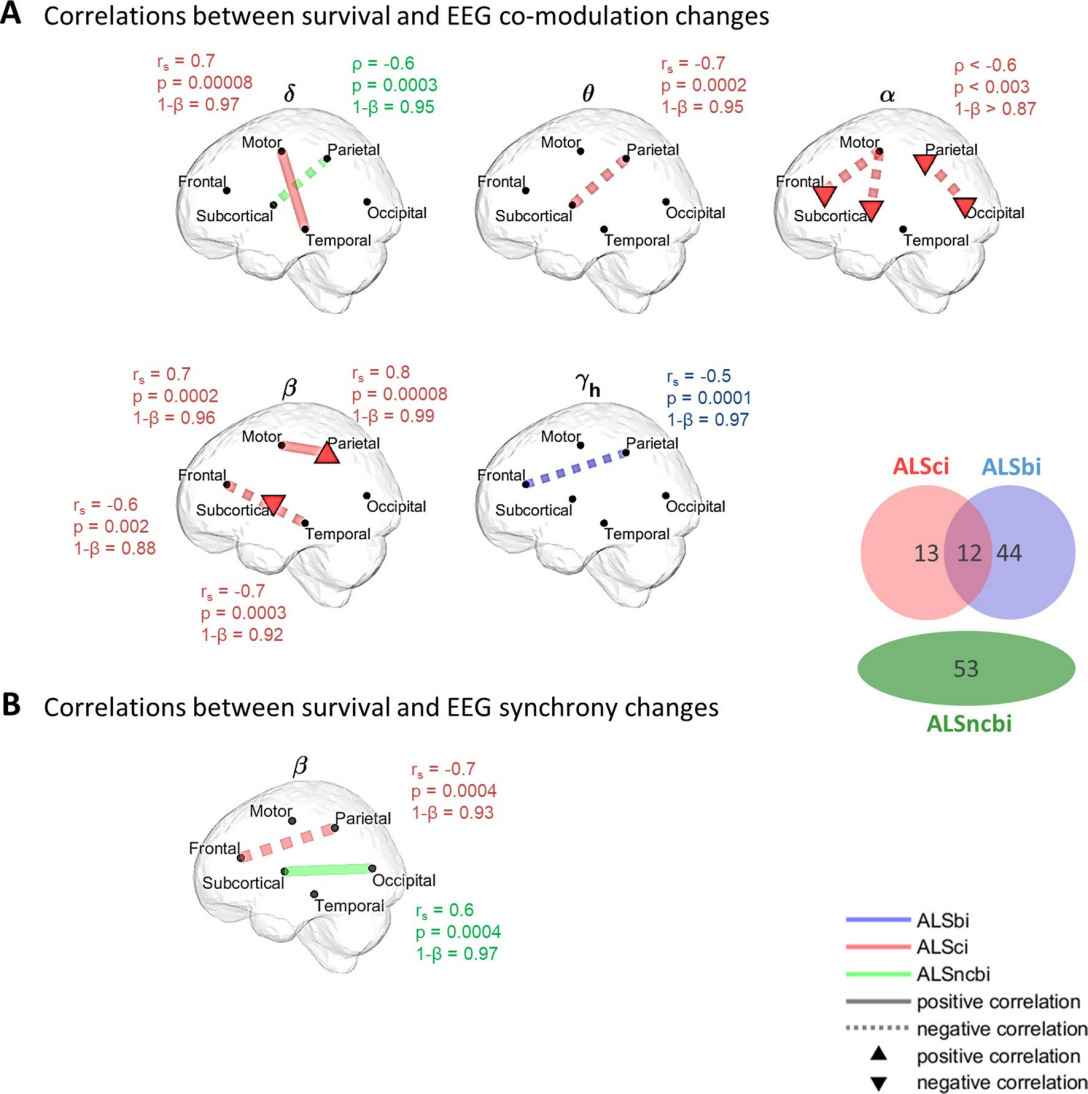
Survival and EEG functional connectivity in ALSci, ALSbi and ALSncbi subgroups. Regions with significant correlations between survival and participant/ROI-specific connectivity (AEC and iCoh) progressions. Solid lines (or upper triangles) depict positive correlations, indicating that higher rates of functional connectivity change are associated with a better prognosis. In contrast, dashed lines (or lower triangles) represent negative correlations, signifying that higher rates of functional connectivity change are linked to a worse prognosis. In ALSci, ALSbi, and ALSncbi subgroups, the correlation coefficient, $\:{r}_{s}$, the p-value, *p*, and the statistical power,1-*β*, are given for each significant correlation. An adaptive FDR was applied to Spearman’s correlations. ALSci: individuals with ALS and impaired cognition; ALSbi: individuals with ALS and impaired behaviour; ALSncbi: individuals with ALS with normal cognition and behaviour

The relationships between network changes and survival outcomes (median: ~3.5 years) are depicted in Fig. 5. In ALSci participants, higher rates of change in co-modulation over the disease timecourse between the frontal and temporal regions ($\:\beta\:$-band) or between the subcortical and parietal lobes ($\:\theta\:$-band) were associated with poorer prognosis (*p* < .001, FDR at *q* = 0.01). Widespread negative correlations were also observed in the $\:\alpha\:$-band, linking higher rates of change in co-modulation and poorer prognosis (*p* < .01, FDR at *q* = 0.01). On the contrary, higher rates of change between the motor and frontal regions ($\:\delta\:$-band), between the motor and parietal regions or within the parietal lobe ($\:\beta\:$-band) were associated with a better prognosis (*p* < .001, FDR at *q* = 0.01).

ALSbi participants showed a link between higher rates of change of $\:{\gamma\:}_{h}$-band co-modulation between frontal and parietal lobes and poorer prognosis (*p* < .001, FDR at *q* = 0.01).

In ALSncbi participants, significant negative correlations were observed between the parietal and subcortical lobes (co-modulation, $\:\delta\:$-band). Positive correlations with survival were found between the subcortical lobe and the occipital area ($\:\beta\:$-iCoh).

**Fig. 6:**
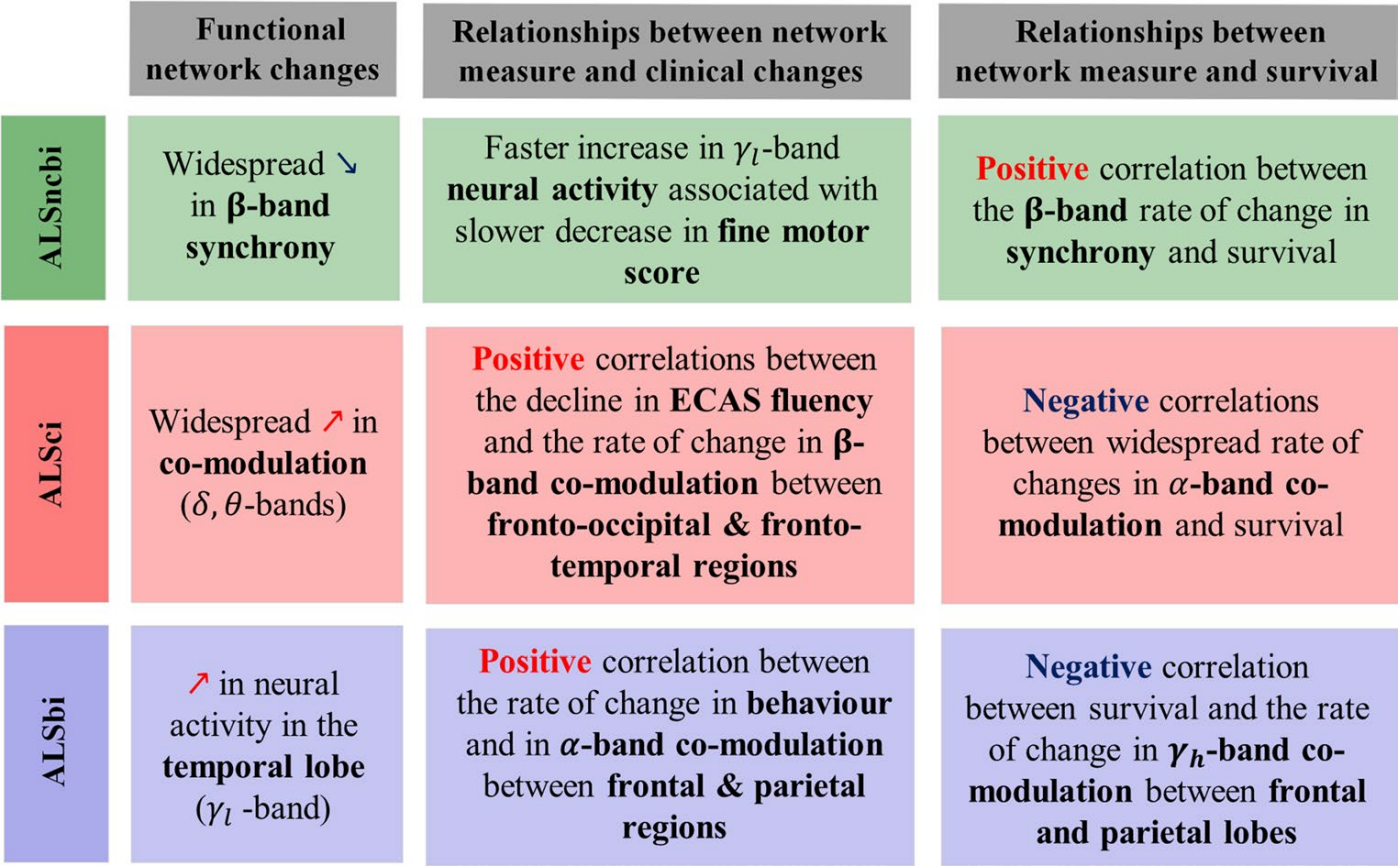
Summary of the main findings. Distinct changes in functional networks characterise cognitive phenotypes in ALS and are clinically relevant. Positive correlations, indicates that higher rates of functional connectivity change are associated with a better prognosis/increased rate of change in cognition or behaviour. In contrast, negative correlations, signify that higher rates of functional connectivity change are linked to a worse prognosis/lower rate of change in cognition or behaviour

## Discussion

This study identified significant longitudinal changes in neural activity within the fronto-temporal region in all of the ALS cognitive phenotypes examined. The longitudinal changes in the fronto-temporal region manifested as a decrease in lower frequency bands and an increase in higher frequency bands spectral power. Further investigation of the potential link between the longitudinal frontotemporal changes and cognitive/behavioural impairments indicated that ALS subgroups with different cognitive and behavioural profiles present with very distinct longitudinal effects. In the ALSci subgroup, we observed a widespread increase in co-modulation, which strongly correlated with cognitive decline (|*r*_*s*_*|* > 0.5, 1-$\:\beta\:$ > 0.8). Meanwhile, in the ALSbi subgroup, we found that higher rates of change in co-modulation between the frontal and parietal lobes were associated with increased rates of change in BBI impairment (|*r*_*s*_ | = 0.4, 1-$\:\beta\:$ = 0.9). Notably, ALSncbi displayed a widespread decrease in β-band synchrony over time. Furthermore, within the ALSncbi subgroup, motor decline was linked to changes in neural activity (|*r*_*s*_ | = 0.4, 1-$\:\beta\:$ > 0.8). In all subgroups, survival was strongly associated with the rates of change in functional connectivity between specific brain regions (correlation coefficients |*r*_*s*_| > 0.5, statistical powers 1-*β* > 0.9). The distinct longitudinal EEG patterns observed in the three cognitive/behavioural profiles, along with the correlation of EEG measures to clinical progression, indicate that these EEG measures reflect underlying network-level impairment specific to cognitive, behavioural, and non-cognitive-behavioural domains (Fig. 6).

### Frontotemporal Longitudinal Changes in Neural Activity

Functional networks in the frontotemporal lobe, a key region of atrophy in ALS (Strong et al. 2017; Trojsi et al. 2020), displayed significant changes over time, across all cognitive-behavioural profiles. Decreased $\:\theta\:$-band and increased $\:\gamma\:$-band spectral power was observed over time in the frontotemporal lobe. Lower $\:\theta\:$-band spectral power has been previously observed in the temporal area in ALS compared to HC group (Dukic et al. 2019; Nasseroleslami et al. 2019) (see Table 2). This longitudinal decrease mirrors the decrease in people with ALS compared to healthy individuals (as measured by our previous cross-sectional study). Frontotemporal and frontal-subcortical circuitry are frequently highly impacted in dementias and neuropsychological diseases (Bonelli and Cummings 2007; Neary et al. 1998; Tekin and Cummings 2002). Notably, post-mortem examinations have revealed synapse loss in the prefrontal cortex of people with ALS, which correlated with cognitive decline (Henstridge et al. 2018). As our observed frontotemporal network changes may have been driven by ALSbi or ALSci participants, we then investigated longitudinal effects within each ALSci, ALSbi and ALSncbi subgroup. The next paragraphs provide more specific details on the observed changes within distinct cognitive-behavioural profiles.

**Table 2:** Comparative table between the main cross-sectional and longitudinal results. Increases are represented in red and decreases in blue

Spectral power – neural activity										
	$\:\delta\:$	$\:\theta\:$			$\:\alpha\:$			$\:\beta\:$	$\:{\gamma\:}_{l}$	$\:{\gamma\:}_{h}$
Cross-sectional (ALS vs. HC) (Dukic et al. 2019)	↘ temporal and posterior regions									
Longitudinal (all ALS)		↘ frontal, temporal regions							↗ frontal and temporal regions	↗ frontal and temporal regions
Co-modulation										
	$\:\delta\:$	$\:\theta\:$				$\:\alpha\:$		$\:\beta\:$	$\:{\gamma\:}_{l}$	$\:{\gamma\:}_{h}$
Cross-sectional (ALS vs. HC) (Dukic et al. 2019)	↗ frontal, central, posterior regions	↗ central, posterior regions							↗ frontal, central, posterior regions	
Longitudinal (all ALS)		Widespread ↗								
Longitudinal ALSci	Widespread ↗					↗ frontal region		Widespread ↗		
Longitudinal ALSncbi	↗ posterior region									
Synchrony										
	$\:\delta\:$	$\:\theta\:$		$\:\alpha\:$			$\:\beta\:$		$\:{\gamma\:}_{l}$	$\:{\gamma\:}_{h}$
Cross-sectional results (ALS vs. HC) (Dukic et al. 2019)	↘ frontal, temporal regions						↘ sensorimotor network: central, temporal regions			
Longitudinal ALSncbi							Widespread ↘			

### Cognitive Impairment and Longitudinal Increase in EEG Co-Modulation

We observed no significant longitudinal changes in neural activity (spectral power) in ALSci subgroup, despite previous cross-sectional studies reporting decreased spectral power in temporo-posterior regions (all ALS group versus HC) (Dukic et al. 2019). Our results, focusing on the ALSci subgroup, do not contradict previous findings; rather, they provide additional information by revealing distinct results within this specific subset, emphasising the importance of subgroup analysis. ALSci participants demonstrated widespread increases in $\:\delta\:$-, $\:\theta\:$- and $\:\beta\:$-band co-modulation over time, which is consistent with previous cross-sectional findings of higher co-modulation in ALS compared to HC in $\:\delta\:$, $\:\theta\:$ and $\:{\gamma\:}_{l}$ frequency bands (Dukic et al. 2019) (Table 2). This longitudinal increase is, again, mirroring the previously observed cross-sectional increase in ALS compared to HC. Burgh et al. similarly observed longitudinal structural connectivity changes in participants with impaired cognition (Burgh et al., 2020). This widespread increase in functional connectivity observed in our study could appear antithetical to the findings of structural atrophy and metabolic reduction at rest in ALS (Kew et al. 1993; Verstraete et al. 2010, 2014). However, approaches integrating structural and functional imaging showed increased functional connectivity within atrophied regions (Douaud et al. 2011; Nasseroleslami et al. 2019; Proudfoot et al. 2018). This increasing connectivity could be explained as compensation for decreasing structural connectivity tracts along with a progressive loss of GABA-ergic inhibitory interneurons and disinhibition of remaining glutamatergic tracts (Douaud et al. 2011; Lloyd et al. 2000). Such disinhibition and hyperexcitability is evidenced by numerous histopathological, neurophysiological, neuroimaging and clinical studies (Turner and Kiernan 2012).

In ALSci, higher rates of connectivity changes in the frontotemporal and fronto-occipital areas (in the β-band) were associated with a more rapid decline in verbal fluency. Verbal fluency deficits are a well-documented cognitive impairment in ALS, frequently reported in previous studies (Abrahams et al. 2000, 2014; Beeldman et al. 2016). These deficits are thought to be mediated by frontotemporal areas (Baldo et al. 2006). By contrast, higher rates of connectivity changes involving motor or parietal regions (part of the sensorimotor network) were linked to a decreased rate of cognitive decline (as measured by the total ECAS score). This inverse relationship suggests a dissociation between connectivity changes involving sensorimotor regions and those involving other brain regions in individuals with ALS who experience cognitive impairment. Individuals with ALS who experience a faster decline in cognition are more likely to exhibit rapid network changes in the fronto-temporal and fronto-occipital regions but are less likely to exhibit rapid network changes in the sensorimotor area.

### Temporal Longitudinal Changes in Behaviourally Impaired Individuals with ALS

In ALSbi, an increase in $\:\gamma\:$-band power was observed in the temporal lobe. These findings are supported by observations of structural cerebral changes in frontotemporal regions of behaviourally impaired participants with ALS (Burgh et al., 2020; Lulé et al. 2018), but have not been reported before in RS-EEG. Furthermore, higher rates of change in co-modulation between the frontal and the parietal lobes were observed in participants with ALS that exhibited a faster decline in BBI scores. This suggests a meaningful connection between alterations in brain connectivity patterns and the progression of behavioural changes in ALS. In the combined ALSci/ASLbi group, the connections identified between EEG functional connectivity and cognitive-behavioural scores highlight the potential of EEG measures as a quantitative marker for disruptions in cognitive and behavioural networks in ALS.

### Motor and extra-motor Functional Changes in Cognitively and Behaviourally Unaffected Participants

In ALSncbi, we observed significant longitudinal changes in spectral power, specifically in the $\:{\gamma\:}_{l}$-band, localized to the frontal lobe. This suggests that cerebral changes extend beyond the primary motor cortex, a phenomenon previously linked to disease progression in the broader ALS population (McMackin et al. 2021; Menke et al. 2018). Evidence from diffusion tensor imaging has indicated a loss of structural connectivity spreading from motor regions to frontoparietal lobes (Verstraete et al. 2014), reinforcing the idea that ALS-related changes propagate from the primary motor cortex to other brain regions. In ALSncbi, we also noted an increase in connectivity (co-modulation) between the fronto-sensorimotor regions and other regions, particularly in the δ and θ frequency bands, aligning with the hypothesis of a progressive spread of ALS-related changes beyond the primary motor cortex.

Additionally, we observed a widespread decrease in β-band synchrony over time in ALSncbi, consistent with previous cross-sectional findings of reduced β-band synchrony in people with ALS (Table 2). This cross-sectional decrease in synchrony correlated with motor impairment and cortical atrophy, further highlighting its clinical relevance.

To confirm the clinical significance of our observations regarding fronto-temporo-parietal changes over time, we correlated them with changes in clinical scores. Associations between EEG data and clinical measures were detected at the whole-brain level, not limited to specific brain regions. The correlations between spectral power and fine motor scores suggest the potential of EEG measures as a prognostic biomarker for motor decline in the ALSncbi group.

Longitudinal and cross-sectional (Table 2) (Dukic et al. 2019) changes in co-modulation were more closely associated with changes in neuropsychological scores, while alterations in synchrony were linked with differences in motor function. Amplitude-coupling (co-modulation) and phase-coupling (synchrony) in EEG and fMRI can provide complementary information on brain function, as they do not quantify exactly the same phenomena and potentially reflect distinct neurobiological mechanisms (Daffertshofer et al. 2018; Mostame and Sadaghiani 2020; Siems and Siegel 2020; Wirsich et al. 2021). Amplitude-coupling may stem from neuromodulation by neuropeptides like norepinephrine or dopamine (van den Brink et al. 2019), whereas phase-coupling appears more relevant in terms of interregional communication during stimuli or cognitive processes (Landau et al. 2015; Nicolaou et al. 2017). Changes in amplitude- and phase-coupling have been observed in neurological conditions such as Alzheimer’s disease or multiple sclerosis, while Parkinson’s disease primarily exhibits alterations in phase-coupling (Engel et al. 2013). Altogether with our observation that alterations in co-modulation and synchrony show distinct associations with cognitive and motor functions, this suggests that combining these EEG functional connectivity measures can offer a more comprehensive assessment of changes in brain function.

### Associations between Functional Connectivity and Survival in ALS Subgroups

In all ALS cognitive-behavioural profiles (ALSci, ALSbi, ALSncbi), the rates of change in both types of functional connectivity showed robust associations with survival, as indicated by high correlation coefficients (|*r*_*s*_| > 0.5) and strong statistical power (1-$\:{\beta\:}_{0.01}$ > 0.9).

Within the ALSncbi group, we observed that an increased rate of change in α-band synchrony between the subcortical and occipital areas was linked to extended survival. Conversely, an increased rate of change in δ-band co-modulation between the subcortical and sensorimotor network was associated with shorter survival. This implies that changes in connectivity within non-motor regions, beyond central and parietal areas, might indicate cerebral compensation that slows disease progression. This highlights enhanced plasticity as a potential focus for future treatment research.

However, we also noted that increased rates of changes in frontotemporal and frontoparietal connectivity ($\:\beta\:$-band) were associated with a less favourable prognosis in participants with cognitive or behavioural impairments. In contrast, increased rate of changes in connectivity within the sensorimotor network ($\:\delta\:$- and $\:\beta\:$-bands) in ALSci were linked to longer survival. These findings imply the coexistence of distinct mechanisms contributing to either a faster or slower progression of the disease. Moreover, these mechanisms appear to vary among ALS cognitive-behavioural profiles.

Individuals with cognitive and behavioural impairments generally have a poorer prognosis compared to those without these impairments. Studies have shown that the presence of these deficits is associated with a faster rate of disease progression (Elamin et al. 2013; Nguyen et al. 2021; Xu et al. 2017). Cognitive and behavioral impairments reflect a broader neurodegenerative process that affects both motor and non-motor regions of the brain. This extensive neurodegeneration could contribute to the overall faster progression of ALS and to the distinct changes in EEG functional connectivity.

Additional neurobiological, genetic and other factors are likely to contribute to the survival and to our spectral EEG measures that might not have been captured in our findings on EEG-Survival relationships.

### Limitations and Considerations in Longitudinal EEG Studies for ALS

A limitation of this study is the attrition in repeated longitudinal recordings, with only 7 out of 124 participants attending the 5th session. Longitudinal changes can be more challenging to detect than cross-sectional differences due to the subtlety of measurements over time compared to the pronounced differences between the ALS and HC groups. While linear mixed-effects models can help account for missing data points, they do not eliminate potential bias. In this study, we assumed that missing recordings were missing at random, although they could be dependent on disease progression and therefore associated with EEG measures. In future research, obtaining longitudinal recordings from both ALS and HC groups would allow for distinguishing between changes resulting from the disease and those associated with test-retest variance and normal aging.

Additionally, the categorisation into ALSci, ALSbi, and ALSncbi would ideally be based on a full neuropsychological assessment rather than on ECAS and BBI scores alone. Furthermore, while ECAS fluency scores serve as a valid measure of verbal fluency, the ECAS is a screening task with reduced sensitivity and specificity compared to full-battery tasks when assessing cognitive impairment (Pinto-Grau et al. 2017).

The lack of information on non-invasive ventilation (NIV) use is another limitation for the analysis of survival in ALS. NIV is a well-established intervention that significantly improves survival by supporting respiratory function (Bourke et al. 2006). Participants using NIV may differ systematically from those who do not in terms of disease rate of progression. To address this limitation, future analyses should include detailed data on NIV use and consider its effect on survival.

## Conclusion

This study demonstrated significant longitudinal changes in neural activity within the frontotemporal region among ALS patients. We have also delineated the progression profiles of spectral EEG measures in distinct ALS subgroups, such as ALSci, ALSbi, and ALSncbi, yielding critical insights into the relationship between these neural activity changes and cognitive or behavioural impairments. Notably, the link between spectral EEG changes over the course of the disease and survival across all subgroups highlights the potential clinical relevance of our findings. These results contribute to untangling the intricate interplay among neural activity alterations, cognitive-behavioural profiles, and ALS progression. We have identified both general and phenotype-specific progression profiles for spectral EEG measures. The specific connections between spectral EEG measures and clinical metrics (ECAS, BBI, and ALSFRS-R) have further validated the ability of EEG measures to quantify and monitor network-level impairments related to cognitive-behavioural deficits. This suggests that distinct mechanisms may underlie variations in disease progression rates among ALS cognitive-behavioural profiles.

Given the strong consistency between the direction of longitudinal changes and the previously observed abnormal EEG signatures in cross-sectional analysis, these measures emerge as robust biomarker candidates for phenotyping, stratification, and tracking the progression of the disease and associated cognitive impairments in clinical trials.

Future research should focus on the intricate dynamics of neural activity changes in ALS to unlock more fine-grained measures for personalised approaches in clinical trials and treatment strategies.

## Electronic Supplementary Material

Below is the link to the electronic supplementary material.

Supplementary Material 1

## Data Availability

The data supporting the findings presented above are available from the corresponding author on reasonable request from qualified investigators. Data sharing is subject to the participant’s consent and approvals by the Data Protection Officer and the Office of Corporate Partnership and Knowledge Exchange in Trinity College Dublin.
