# Supplementary material for: Distinct Longitudinal Changes in EEG Measures Reflecting Functional Network Disruption in ALS Cognitive Phenotypes
Source: Brain Topogr. 2024 Oct 4;38(1):3. doi: 10.1007/s10548-024-01078-8 (PMC11452478; doi:10.1007/s10548-024-01078-8)
Supplement: Supplementary file 1 — Supplementary Material 1 [file 10548_2024_1078_MOESM1_ESM.docx]

Appendix – Supplementary materials

## Supplementary note 1: Brain networks

To define neurophysiologically-meaningful networks, we used the five anatomical lobes: frontal, temporal, centro-parietal, occipital and subcortical with the separation of the centro-parietal lobe into parietal and motor networks as this last brain region is a hallmark of atrophy in ALS. Each network is described in terms of the AAL regions in Table 1.

*Table 1: Subgroups of brain regions according to the AAL atlas.*

| **Motor network** | Precentral gyrus,  Rolandic operculum,  Supplementary motor area,  Paracentral lobule |
| --- | --- |
| **Frontal lobe** | Superior frontal gyrus, dorsolateral, Superior frontal gyrus, orbital, Middle frontal gyrus, Middle frontal gyrus, orbital, Inferior frontal gyrus, opercular, Inferior frontal gyrus, triangular, Inferior frontal gyrus, orbital, Superior frontal gyrus, medial, Superior frontal gyrus, medial orbital, Gyrus rectus |
| **Temporal lobe** | Fusiform gyrus, Heschl gyrus, Superior temporal gyrus, Temporal pole: superior temporal gyrus, Middle temporal gyrus, Temporal pole: middle temporal gyrus Inferior temporal gyrus, |
| **Occipital lobe** | Calcarine fissure and surrounding cortex, Cuneus, Lingual gyrus, Superior occipital lobe, Middle occipital lobe, Inferior occipital lobe |
| **Parietal lobe** | Postcentral gyrus, Superior parietal gyrus, Inferior parietal gyrus, Supramarginal gyrus, Angular gyrus, Precuneus, |
| **Subcortical** | Amygdala,  Caudate nucleus,  Cingulate gyrus, anterior part, Cingulate gyrus, mid part,  Cingulate gyrus, posterior part,  Hippocampus,  Insula,  Olfactory cortex,  Pallidum,  Parahippocampus, Putamen, Thalamus |

## Supplementary note 2: Longitudinal models of EEG spectral measures

Including a large number of covariates in the model can increase its complexity and the risk of overfitting. Overfitting can lead to models that are too tailored to a specific dataset, reducing their generalisability to other populations. We carefully tailored the parameters to create more robust and generalisable models. The primary aim of the study was to analyse how distinct cognitive-behavioural profiles in ALS manifest unique patterns of longitudinal EEG spectral measures. We therefore performed stratified analyses for cognitive/behaviour impairment to investigate neural network disruptions in relation to cognitive phenotypes (ie. independents LME models per cognitive-behavioural profile).

The LME models allow for the inclusion of all available data points and can account for individual differences in baseline measures and trajectories over time. Each participant can have their own intercept and slope, capturing how different individuals experience changes over time. The EEG spectral measures progressions over time were estimated as follows, in Wilkinson notation:

EEG Measure ~ Time + (Time|Participant)

Age and gender were considered for inclusion. As we specifically looked into the progressions of EEG measures over time, we were interested in the longitudinal effect of age or gender on EEG measures. The following model was considered:

EEG Measure ~ Time + (Time|Participant) + (Time|Age) + (Time|Gender)

In each case (each EEG measure, frequency band and ROI), a likelihood ratio test was applied to compare models with or without the age and gender effects. The simpler models (without age/gender) were significantly better (q > .05) than the ones with age or gender as random-effects. *Spectral power*

For each frequency band of the spectral power with ROI-specific significant changes over time (significance determined by bootstrapping analysis on the LME models per ROI), the F-tests were computed with the null hypothesis $H_{0}$ of all fixed-effects being null. The corresponding F-statistics can be observed in Table 2.

*Table 2: Spectral power model F-statistics performed for each frequency band of interest. Each rejected null hypothesis (p<0.05) reveals the existence of non-zero fixed-effects.*

|  | All | | ALSbi | | ALSncbi |
| --- | --- | --- | --- | --- | --- |
| Frequencies | δ | γ_l_ | $\gamma_{l}$ | $\gamma_{h}$ | $\beta$ |
| p-values | 0.2 | 0.2 | 0.2 | 0.2 | 0.5 |
| F-statistics | 1.8 | 1.5 | 1.5 | 1.6 | 0.5 |

For each frequency-band model of interest, the fixed-effects and their related statistics are detailed in Table 3. It describes the estimated effects of time (since disease onset) on the EEG power. Depending on the frequency band, the effects differ, but no overall significant temporal effect was observed in any model.

*Table 3: Spectral power models fixed-effects and related t-statistics for each frequency band of interest. For each fixed-effect, is given the estimate and its standard error as well as the t-statistic with p-value and confidence intervals. Significant effects are represented in bold. No significant overall time effect was observed for any of the six frequency-band.*

|  | Freq | Fixed-effect name | Estimate | SE | tStat | pValue | Lower | Upper |
| --- | --- | --- | --- | --- | --- | --- | --- | --- |
| All | $\theta$ | **(Intercept)** | **0.082** | **0.0073** | **11** | **2E-29** | **0.068** | **0.096** |
|  |  | Time | -0.0003 | 0.0002 | -1.3 | 0.18 | -0.0007 | 0.0001 |
|  | $\gamma_{l}$ | **(Intercept)** | **0.1** | **0.012** | **8.9** | **6E-19** | **0.081** | **0.13** |
|  |  | Time | 0.00033 | 0.00027 | 1.2 | 0.22 | -0.0002 | 0.00085 |
| ALSbi | $\boldsymbol{\gamma}_{\boldsymbol{l}}$ | **(Intercept)** | **0.095** | **0.014** | **6.7** | **2E-11** | **0.067** | **0.12** |
|  |  | Time | 0.00061 | 0.0005 | 1.2 | 0.22 | -0.0004 | 0.0016 |
|  | $\boldsymbol{\gamma}_{\boldsymbol{h}}$ | **(Intercept)** | **0.17** | **0.032** | **5.2** | **2E-07** | **0.1** | **0.23** |
|  |  | Time | 0.0015 | 0.0011 | 1.3 | 0.2 | -0.0008 | 0.0037 |
| ALSncbi | $\boldsymbol{\gamma}_{\boldsymbol{l}}$ | **(Intercept)** | **0.11** | **0.015** | **7.2** | **7E-13** | **0.078** | **0.14** |
|  |  | Time | 0.0002 | 0.0003 | 0.71 | 0.48 | -0.0004 | 0.0007 |

*Amplitude envelope correlation (AEC)*

For each frequency band with ROI-specific significant AEC changes over time, the F-tests were computed with the null hypothesis $H_{0}$ of all fixed-effects being null. The corresponding F-statistics can be observed in Table 4.

*Table 4: Co-modulation model F-statistics performed for each frequency band of interest. Each rejected null hypothesis (p<0.05) reveals the existence of non-zero fixed-effects other than the intercept.*

|  | ALSci | | | | ALSbi | ALSncbi | |
| --- | --- | --- | --- | --- | --- | --- | --- |
| Frequencies | **δ** | **θ** | α | **β** | $\alpha$ | $\delta$ | $\theta$ |
| p-values | **0.0002** | **0.00003** | 0.4 | **0.04** | 0.7 | 0.2 | 0.09 |
| F-statistic | **14** | **17** | 0.84 | **4.1** | 0.16 | 1.5 | 2.8 |

For each frequency-band model, the fixed-effects and their related statistics are detailed in Table 5. It describes the estimated effects of time (since disease onset) on the EEG power. Depending on the frequency band, the effects differ but overall significant temporal effects were observed in the $\delta$ and $\theta$-bands, in ALSci patients.

*Table 5: Co-modulation models fixed-effects and related t-statistics for each frequency band of interest. For each fixed-effect, is given the estimate and its standard error as well as the t-statistic with p-value and confidence intervals. Significant effects are represented in bold.*

|  | Freq | Fixed-effect name | Estimate | SE | tStat | pValue | Lower | Upper |
| --- | --- | --- | --- | --- | --- | --- | --- | --- |
| **ALSci** | $\boldsymbol{\delta}$ | **(Intercept)** | **-1.9** | **0.58** | **-3.2** | **0.0013** | **-3** | **-0.73** |
|  |  | **Time** | **0.074** | **0.02** | **3.7** | **0.0002** | **0.035** | **0.11** |
|  | $\boldsymbol{\theta}$ | **(Intercept)** | **-1.1** | **0.33** | **-3.5** | **0.0005** | **-1.8** | **-0.51** |
|  |  | **Time** | **0.051** | **0.01** | **4.1** | **3E-05** | **0.027** | **0.075** |
|  | $\boldsymbol{\alpha}$ | (Intercept) | -0.052 | 0.48 | -0.1 | 0.91 | -0.98 | 0.88 |
|  |  | Time | 0.015 | 0.02 | 0.92 | 0.36 | -0.017 | 0.046 |
|  | $\boldsymbol{\beta}$ | **(Intercept)** | **-0.5** | **0.42** | **-1.2** | **0.23** | **-1.3** | **0.32** |
|  |  | **Time** | **0.034** | **0.02** | **2** | **0.041** | **0.002** | **0.067** |
| **ALSbi** | $\boldsymbol{\alpha}$ | (Intercept) | 0.16 | 0.34 | 0.47 | 0.64 | -0.5 | 0.82 |
|  |  | Time | -0.0053 | 0.01 | -0.4 | 0.69 | -0.03 | 0.021 |
| **ALSncbi** | $\boldsymbol{\delta}$ | (Intercept) | -0.048 | 0.5 | -0.096 | 0.92 | -1 | 0.93 |
|  |  | Time | 0.016 | 0.013 | 1.2 | 0.23 | -0.0097 | 0.041 |
|  | $\boldsymbol{\theta}$ | (Intercept) | -0.4 | 0.5 | -0.79 | 0.43 | -1.4 | 0.58 |
|  |  | Time | 0.025 | 0.015 | 1.7 | 0.094 | -0.0043 | 0.054 |

The estimated longitudinal progressions were plotted for each co-modulation model showing significant longitudinal progression (significant fixed-effect other than the intercept) (Figure 1).


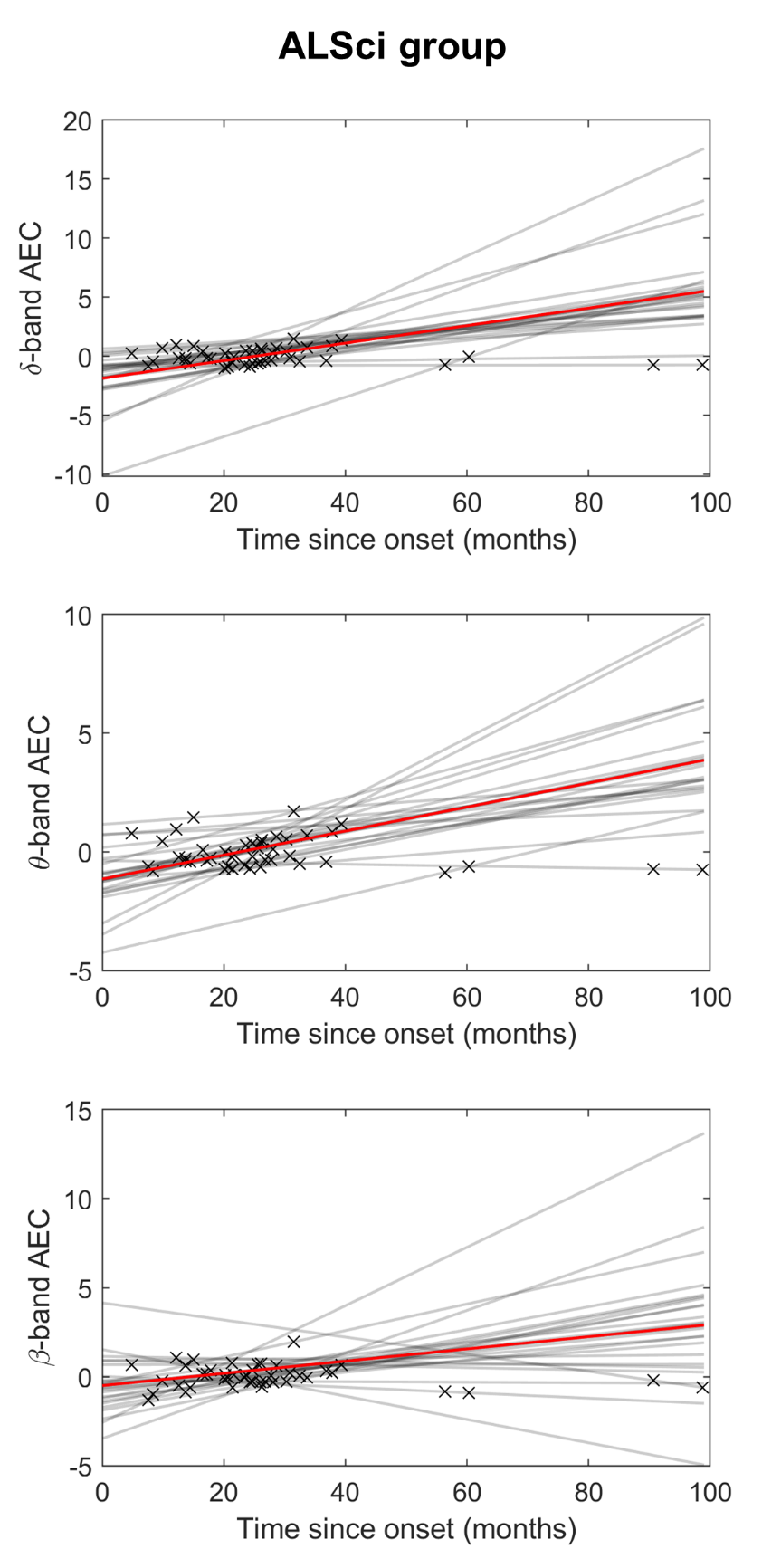


Figure 1: Estimations of the significant (p < .05) longitudinal co-modulation changes for the ALSci group. Grey lines represent linear models of AEC change per participant (based on random-effects), while red lines represent the overall linear changes (based on fixed-effects). Crosses represent recording times for each participant.

*Imaginary coherence (iCoh)*

Significant fixed-effects were observed for non-cognitively impaired patients in $\beta$-band, in the synchrony model (Table 6).

*Table 6: Synchrony model F-statistics performed for each frequency band of interest. Each rejected null hypothesis (p<0.05) reveals the existence of non-zero fixed-effects.*

|  | ALSci | ALSbi | | ALSncbi | | |
| --- | --- | --- | --- | --- | --- | --- |
| Frequencies | $\beta$ | $\delta$ | $\alpha$ | $\delta$ | $\boldsymbol{\beta}$ | γ_h_ |
| p-values | 0.9 | 0.2 | 0.4 | 0.1 | **0.003** | 0.2 |
| F-statistic | 0.03 | 1.9 | 0.7 | 2.3 | **8.8** | 1.4 |

For each frequency-band model, the fixed-effects and their related statistics are detailed in Table 7. It describes the estimated effects of time (since disease onset) on the EEG power. Depending on the frequency band, the effects differ, but whole brain $\beta$-band synchrony significantly decreased over time in ALSncbi patients.

*Table 7: Synchrony models fixed-effects and related t-statistics for each frequency band of interest. For each fixed-effect, is given the estimate and its standard error as well as the t-statistic with p-value and confidence intervals. Significant effects are represented in bold. A significant overall time effect was observed in* $\beta$*-band for ALSncbi patients.*

|  | Freq | Fixed-effect name | Estimate | SE | tStat | pValue | Lower | Upper |
| --- | --- | --- | --- | --- | --- | --- | --- | --- |
| **ALSci** | $\boldsymbol{\beta}$ | (Intercept) | -0.048 | 0.2 | -0.2 | 0.81 | -0.44 | 0.34 |
|  |  | Time | 0.0017 | 0.01 | 0.17 | 0.86 | -0.02 | 0.021 |
| **ALSbi** | $\boldsymbol{\delta}$ | (Intercept) | -0.27 | 0.21 | -1.3 | 0.2 | -0.69 | 0.14 |
|  |  | Time | 0.0067 | 0.006 | 1.1 | 0.29 | -0.006 | 0.019 |
|  | $\boldsymbol{\alpha}$ | (Intercept) | -0.15 | 0.18 | -0.9 | 0.4 | -0.51 | 0.2 |
|  |  | Time | 0.0046 | 0.005 | 0.85 | 0.39 | -0.006 | 0.015 |
| **ALSncbi** | $\boldsymbol{\delta}$ | **(Intercept)** | **-0.3** | **0.14** | **-2.2** | **0.028** | **-0.57** | **-0.033** |
|  |  | Time | 0.0074 | 0.005 | 1.5 | 0.13 | -0.002 | 0.017 |
|  | $\boldsymbol{\beta}$ | **(Intercept)** | **0.45** | **0.16** | **2.8** | **0.005** | **0.14** | **0.77** |
|  |  | **Time** | **-0.013** | **0.004** | **-3** | **0.003** | **-0.021** | **-0.0043** |
|  | $\boldsymbol{\gamma}_{\boldsymbol{h}}$ | (Intercept) | 0.23 | 0.2 | 1.1 | 0.25 | -0.16 | 0.61 |
|  |  | Time | -0.0054 | 0.005 | -1.2 | 0.23 | -0.014 | 0.0035 |

The estimated longitudinal progressions were plotted for each synchrony (iCoh) model showing significant longitudinal progression (significant fixed-effect other than the intercept) ().


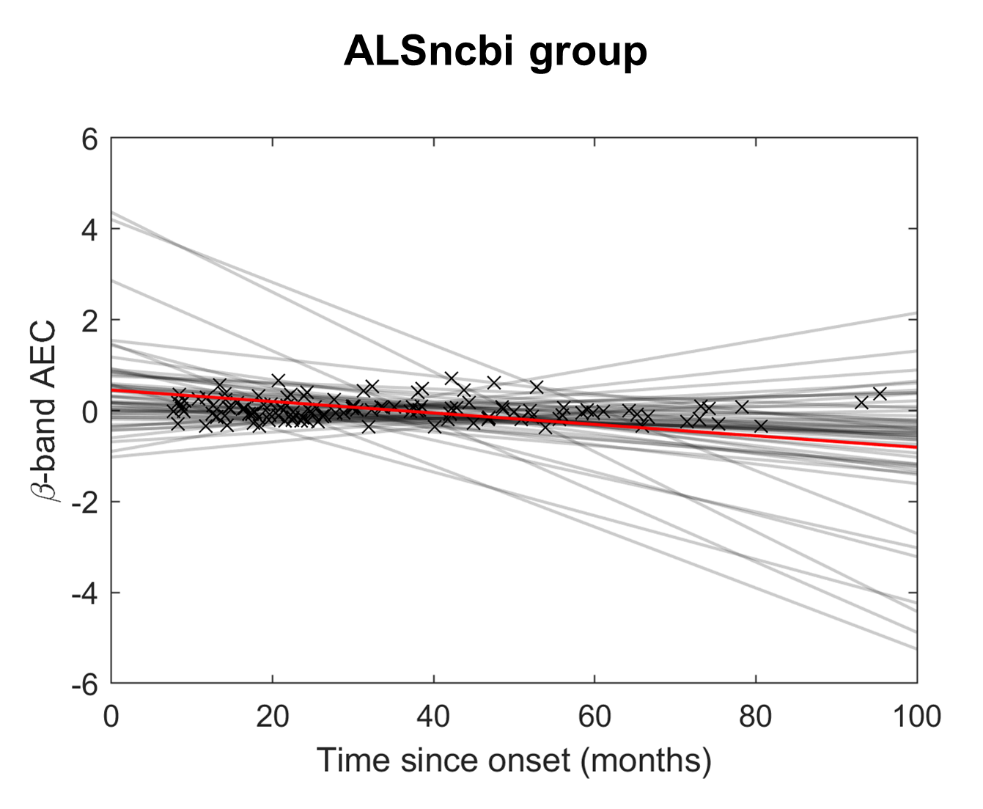


Figure 2: Estimations of the significant (p < .05) longitudinal synchrony changes for the ALSncbi group. Grey lines represent linear models of iCoh change per participant (based on random-effects), while red lines represent the overall linear changes (based on fixed-effects). Crosses represent recording times for each participant.

## Supplementary note 3: Longitudinal models of motor and cognitive clinical measures

We estimated the longitudinal changes in functional clinical scores (ALSFRS-R and neuropsychological scores) using linear mixed-effects models. The goodness-of-fit was estimated using the negative log-likelihood of the fitted model: the lower the value, the best the model fits the dataset. On average across participants, an ALSFRS-R score decreases by 0.72 points per month. The estimated variance of the random slope $\hat{\sigma}_{0}$ was of $0.5$ and that of random intercept $\hat{\sigma}_{1}$ was $8$. The ALSFRS-R subscores and neuropsychology models can be interpreted similarly. For the ECAS scores models questionnaire version fixed-effects and level of education random-effects were additionally estimated.

*Table 8: Longitudinal models of the clinical measures of functional disability and neuropsychology. Fixed- and random-effects of the linear mixed-effects models describing clinical scores (ALSFRS, ECAS, BBI) progressions over the time of the disease. Standard errors were added in parenthesis. *p < 0.05; ***p < 0.001*

|  | | ALSFRS-R | | Neuropsychology | | |
| --- | --- | --- | --- | --- | --- | --- |
|  | | total ALSFRS-R | upper limbs | total ECAS | fluency | BBI |
| Negative log-likelihood | | -3120 | -2107 | -1200 | -950 | -993 |
| *Fixed-effects* | | | | | | |
| Intercept | | 50 (0.8) *** | 12 (0.3) *** | 102 (2) *** | 16 (0.7) *** | 11 (1.6) *** |
| Version B | | - | - | 2 (0.9) | 0.4 (0.4) | - |
| Version C | | - | - | 1 (1) | 0.6 (0.5) | - |
| Time (per months) | | **-0.7 (0.05) ***** | **-0.2 (0.01) ***** | **0.2 (0.07) *** | 0.04 (0.02) | -0.05 (0.05) |
| *Random-effects* | | | | | | |
| Participant | **Intercept variance** | 8 | 3 | 14 | 4 | 12 |
|  | **Time variance** (per months^2^) | 0.5 | 0.2 | 0.2 | 0.01 | 0.07 |
| Education | **Intercept variance** | - | - | 8 | 2 | - |
|  | **Time variance** (per months^2^) |  |  | 0.02 | 0.04 |  |
| Residual | | 2 | 1 | 5 | 2 | 8 |

The linearity of ALSFRS-scores progression has been discussed, with the hypothesis of a curvilinear evolution proposed (Gordon et al., 2010), but a linear regression remains a valid estimation. In our model, while the rate of disease progression was expected to be participant-specific, the variability in the initial ALSFRS-R score across participants was larger than predicted. This may be due to uncertainty in the estimated onset time. Similarly, the ECAS scores (total and fluency) have already been demonstrated to have a linear progression over time (Costello et al., 2021), and our results supported this hypothesis. The ECAS total scores showed a significant increase (p < 0.05), likely caused by the practice effect and by potential non-random dropouts (Costello et al., 2020).

## Supplementary note 4: Checks for potential confounding factors

To evaluate the effect of age, gender or medication on the observed EEG measures progressions, additional statistical tests were performed.

| 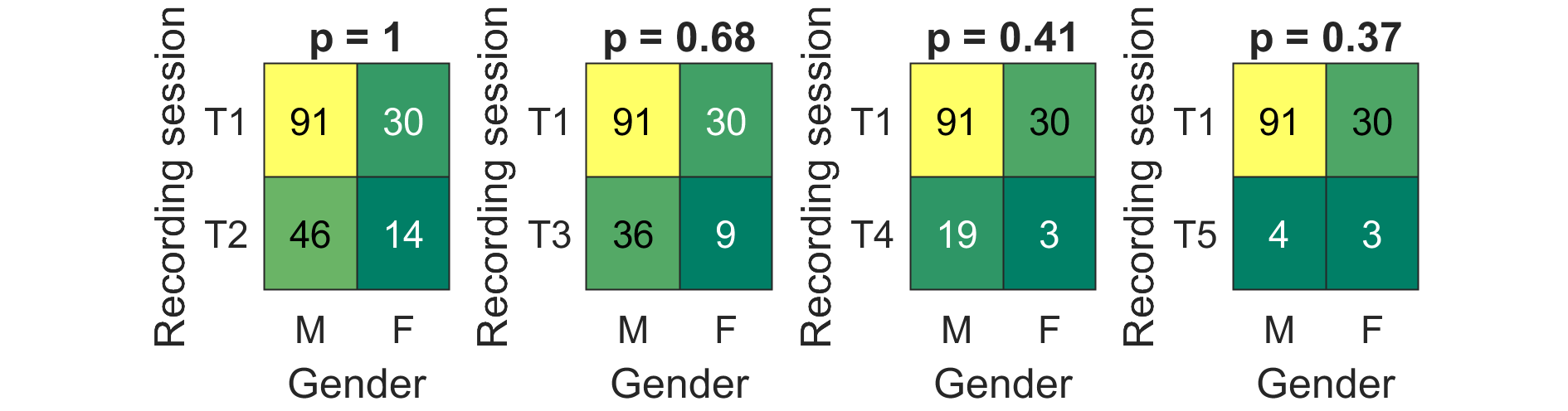   1. *Gender distributions in different recording sessions (T1-T5). Fisher’s exact test (*$\alpha$*=0.05, two-tailed) did not reveal any non-random association between gender and recording sessions.* |
| --- |
| 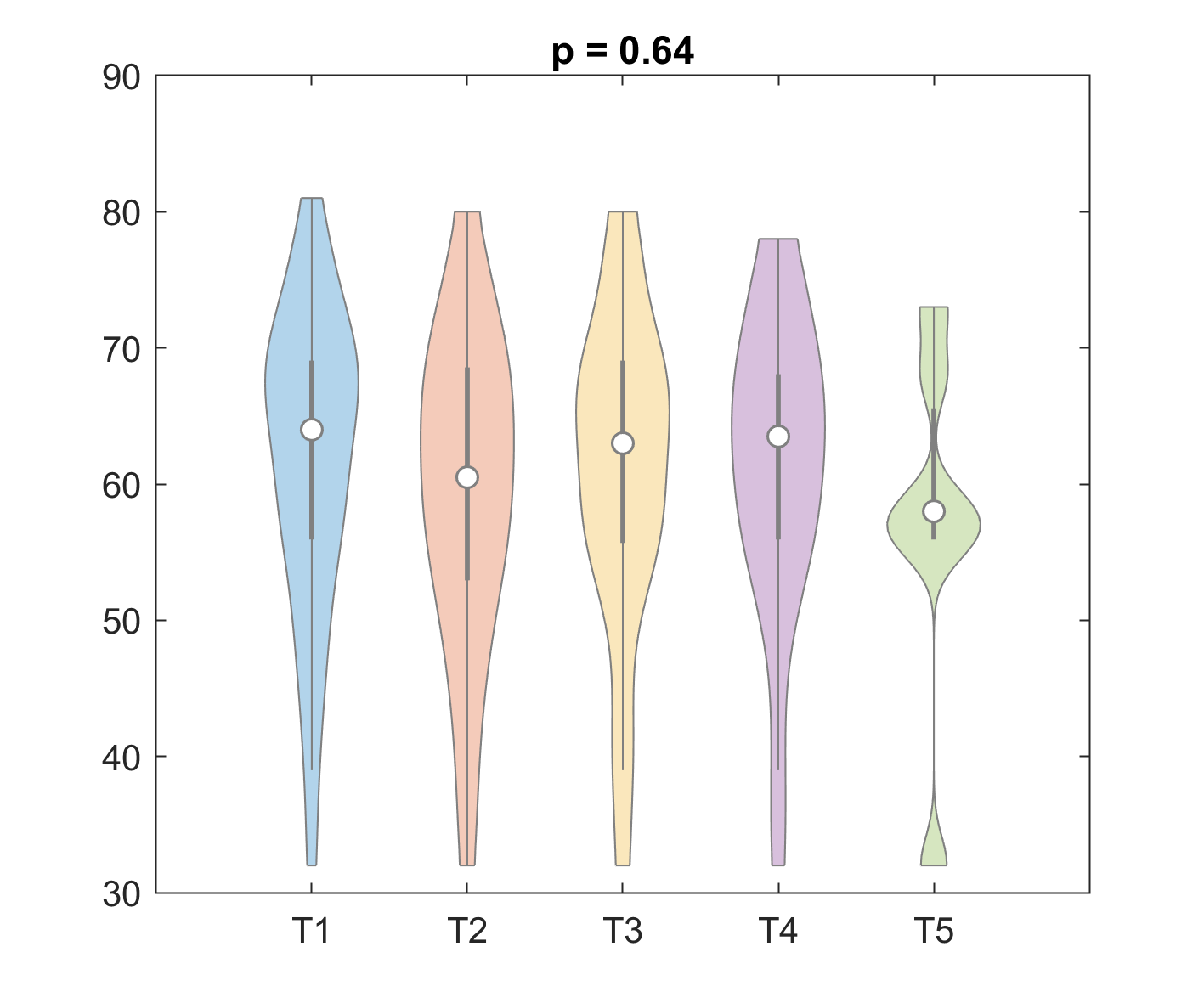   1. *Age distributions in patients' different recording sessions (T1-T5). Kruskal-Wallis's one-way analysis of variance revealed no statistical difference in age distribution between recording sessions.* (Bechtold, 2016/2022) |
| 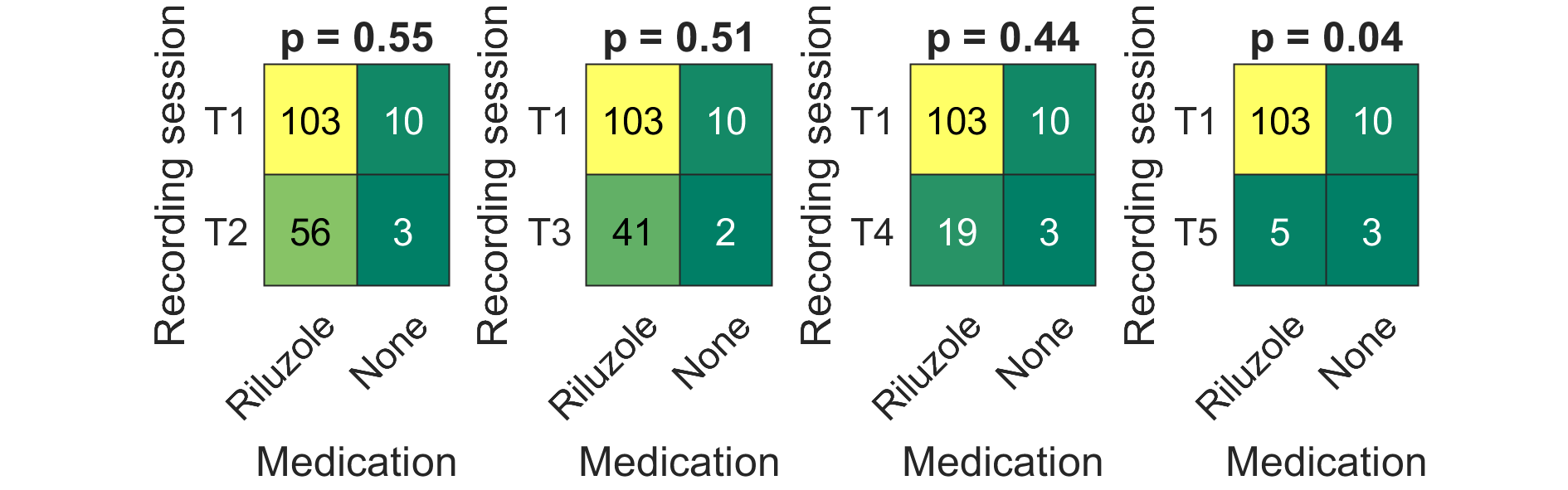  *(c) Medication distributions in different recording sessions (T1-T5). Fisher’s exact test (*$\alpha$*=0.05, two-tailed) did not reveal any non-random association between medication and recording sessions T1 to T4. Unfortunately, the sample size for T5 was too small to be conclusive.* |

## Supplementary note 5: Additional analyses on the linearity of the EEG measures

To test whether the longitudinal changes of the EEG measures can be estimated by a linear model, we applied a quadratic model to the participants with more than two recording sessions (N=37). For each observed longitudinal change, we assessed potential quadratic time effects, (i.e. EEG ~ Time + Time^2^, expressed in Wilkinson notation). For connectivity measures, the inverse normal transformation was used to transform EEG data to a standard normal distribution. The linearity assumption was verified for the majority of the participants (q<.05). In case of a significant quadratic effect, we inspected the patterns of change over time to ensure that despite the non-linearity, the patterns were still monotonic.

## Supplementary note 6: Localisation of significant longitudinal changes of EEG spectral power and functional connectivity in participants with normal and impaired cognition/behaviour


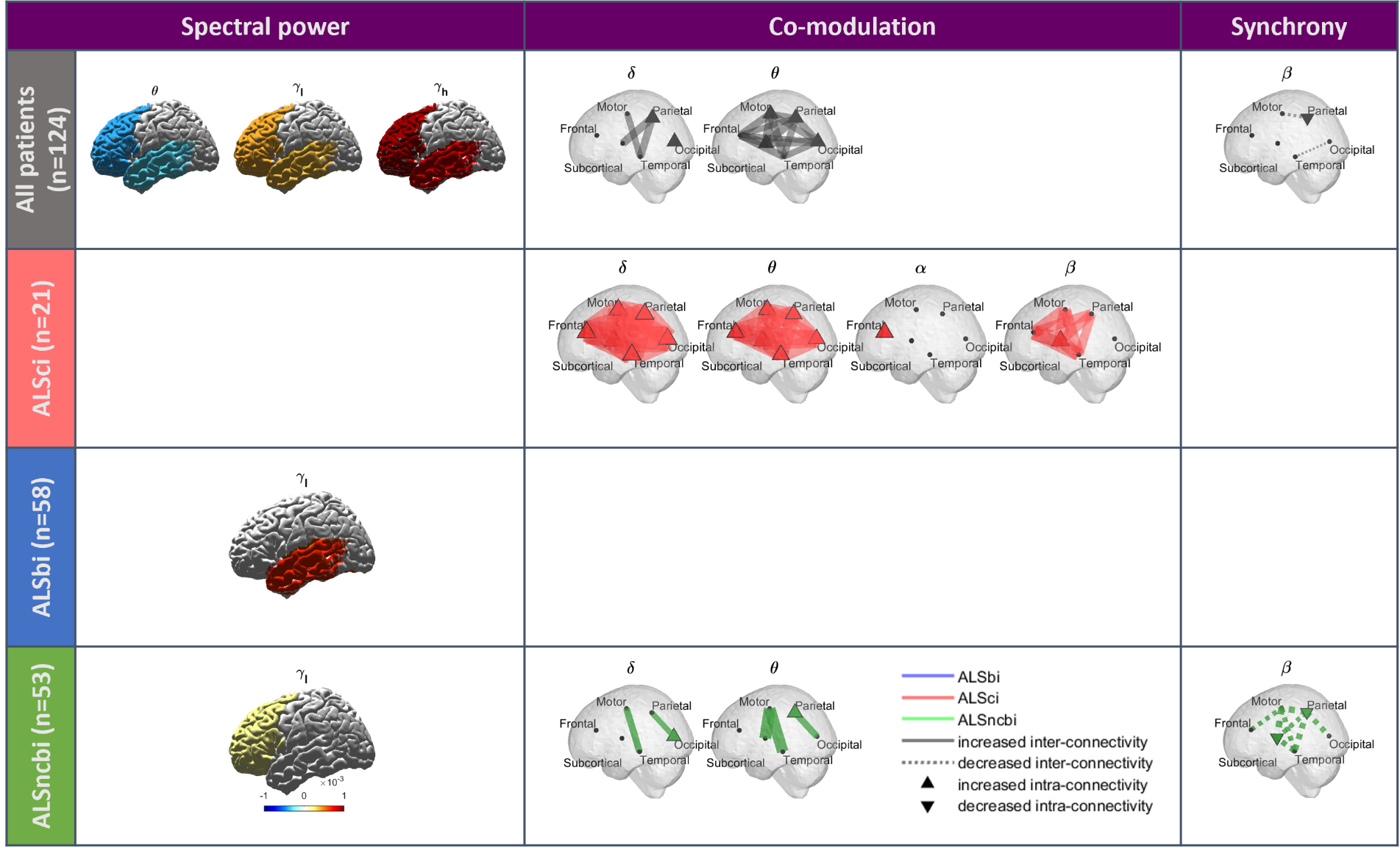
Figure 3: **Left.** Longitudinal changes of EEG spectral power in participants with normal and impaired cognition/behaviour. The significant temporal spectral power variations, in terms of the time fixed-effect and the time ROI-specific random-effects (Bootstrapping, p<0.05), were mapped to get a spatial visualisation. **Middle-Right.** Localisation of longitudinal changes of EEG co-modulation and synchrony in ALS, ALSci, ALSbi and ALSncbi groups. The significant temporal connectivity changes were mapped to get a spatial visualisation of their magnitudes. The temporal variations represent the combined estimated slope (significance by bootstrapping, q<0.1). The dashed lines represent a decrease while the solid lines represent an increase in connectivity. A filled node represents significant intra-lobe connectivity.

## Supplementary note 7: Demographics of the subgroups with distinct cognitive-behavioural profiles – ALSci, ALSbi and ALSncbi

The study included 25 participants in the ALSci subgroup, 58 in the ALSbi and 53 in the ALSncbi. 14 participants were included in both the ALSci and the ALSbi groups. The details of the group demographics can be found in **Table 9**. No significant difference in survival was observed between groups (Kruskal-Wallis; p = 0.3). Participants underwent a maximum of five recording sessions. The distributions across recording times (Figure 2) showed no significant difference between the three subgroups (Kruskal-Wallis; p = 0.2).

**Table 9: Demographic profiles of the neuropsychological subgroups.** For each neuropsychological subgroup, the table details the number of participant per group, the gender proportions (percentage of males), the average ages and disease durations at recording, the survival times and the number of participants who had non-invasive ventilation (NIV) prescribed before their last EEG recording. When applicable, mean and standard deviation are included. ALSci: individuals with ALS and impaired cognition; ALSbi: individuals with ALS and impaired behaviour; ALSncbi: individuals with ALS with normal cognition and behaviour.

| Group | N | M (%) | Age (years) | Disease duration (months) | Survival from symptom onset (years) | NIV before last EEG recording |
| --- | --- | --- | --- | --- | --- | --- |
| **ALSci** | 25 | 80 | 64 ± 9.1 | 23 ± 18 | 4.2 ± 2.2 | 6 |
| **ALSbi** | 58 | 78 | 63 ± 10 | 23 ± 16 | 4.7 ± 2.7 | 15 |
| **ALSncbi** | 53 | 68 | 60 ± 12 | 27 ± 19 | 4.0 ± 2.7 | 10 |


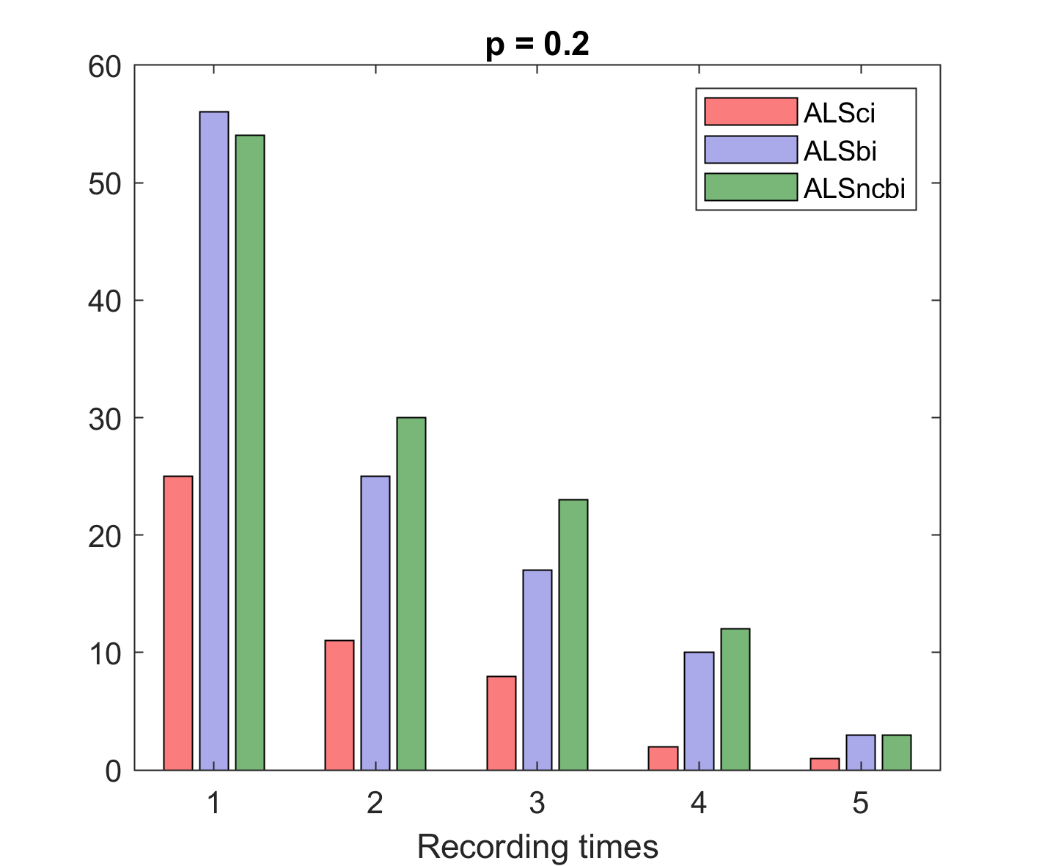


Figure 4: Number of participants in each neuropsychological subgroup for each of the five recording times. The distribution across recording times is not significantly different between the three subgroups (Kruskal-wallis, p = 0.2). ALSci: individuals with ALS and impaired cognition; ALSbi: individuals with ALS and impaired behaviour; ALSncbi: individuals with ALS with normal cognition and behaviour.

## Supplementary note 8: Correlations between functional impairment and EEG functional connectivity in ALSci subgroup.

**Table 10**: **Correlations between ALSFRS-R subscores** **and EEG functional connectivity in ALSci subgroup.** Regions with significant correlations between ALSFRS-R subscores and participant/ROI-specific connectivity (AEC and iCoh) progressions. Positive correlations indicate that higher rates of functional connectivity change are associated with higher decline rates in functional ALSFRS-R subscores. In contrast, negative correlations signify that higher rates of functional connectivity change are linked to lower decline rates in functional impairment. The correlation coefficient, $r_{s}$, the p-value, p, and the statistical power,1-β, are given for each significant correlation. An adaptive FDR was applied to Spearman’s correlations. ALSci: individuals with ALS and impaired cognition.

| **Co-modulation (AEC)** | | | | | |
| --- | --- | --- | --- | --- | --- |
| ALSFRS-R subscores | EEG freq | Brain regions | $r_{s}$ | p-value | statistical power |
| **Bulbar** | $\delta$ | Motor & Temporal | 0.63 | 0.0009 | 0.93 |
|  |  | Subcortical & Occipital | -0.6 | 0.0032 | 0.84 |
|  | $\theta$ | Frontal & Parietal | -0.6 | 0.0032 | 0.8 |
|  |  | Parietal & Subcortical | -0.7 | 0.0004 | 0.94 |
| **Lower limbs** | $\alpha$ | Parietal & Occipital | -0.6 | 0.001 | 0.86 |
|  |  | Parietal & Parietal | -0.6 | 0.002 | 0.87 |
| **Synchrony (iCoh)** | | | | | |
| ALSFRS-R subscores | EEG freq | Brain regions | $r_{s}$ | p-value | statistical power |
| **Bulbar** | $\delta$ | Frontal & Temporal | 0.68 | 0.00028 | 0.97 |
|  | $\beta$ | Frontal & Parietal | -0.61 | 0.0017 | 0.92 |
|  |  | Subcortical & Occipital | -0.67 | 0.00039 | 0.96 |
| **Upper limbs** | $\theta$ | Motor & Subcortical | -0.6 | 0.0019 | 0.85 |
|  |  | Subcortical & Subcortical | 0.64 | 0.00083 | 0.91 |

## References

Bechtold, B. (2022). *Violin Plots for Matlab* [MATLAB]. https://github.com/bastibe/Violinplot-Matlab (Original work published 2016)

Costello, E., Lonergan, K., Madden, C., O’Sullivan, M., Mays, I., Heverin, M., Pinto-Grau, M., Hardiman, O., & Pender, N. (2020). Equivalency and practice effects of alternative versions of the Edinburgh Cognitive and Behavioral ALS Screen (ECAS). *Amyotrophic Lateral Sclerosis and Frontotemporal Degeneration*, *21*(1–2), 86–91. https://doi.org/10.1080/21678421.2019.1701681

Costello, E., Rooney, J., Pinto-Grau, M., Burke, T., Elamin, M., Bede, P., McMackin, R., Dukic, S., Vajda, A., Heverin, M., Hardiman, O., & Pender, N. (2021). Cognitive reserve in amyotrophic lateral sclerosis (ALS): A population-based longitudinal study. *Journal of Neurology, Neurosurgery & Psychiatry*, *92*(5), 460–465. https://doi.org/10.1136/jnnp-2020-324992

Gordon, P. H., Cheng, B., Salachas, F., Pradat, P.-F., Bruneteau, G., Corcia, P., Lacomblez, L., & Meininger, V. (2010). Progression in ALS is not linear but is curvilinear. *Journal of Neurology*, *257*(10), 1713–1717. https://doi.org/10.1007/s00415-010-5609-1
